# Dissecting the Genetic Architecture of Melon Chilling Tolerance at the Seedling Stage by Association Mapping and Identification of the Elite Alleles

**DOI:** 10.3389/fpls.2018.01577

**Published:** 2018-10-31

**Authors:** Juan Hou, Ya-Feng Zhou, Lu-Yin Gao, Yan-Ling Wang, Lu-Ming Yang, Hua-Yu Zhu, Ji-Ming Wang, Sheng-Jie Zhao, Chang-Sheng Ma, Shou-Ru Sun, Jian-Bin Hu

**Affiliations:** ^1^College of Horticulture, Henan Agricultural University, Zhengzhou, China; ^2^Henan Key Laboratory of Fruit and Cucurbit Biology, Zhengzhou, China; ^3^Zhengzhou Fruit Research Institute, Chinese Academy of Agricultural Sciences, Zhengzhou, China

**Keywords:** melon, seedling stage, chilling tolerance, genetic architecture, association mapping, elite allele

## Abstract

Low temperature is an important abiotic stress that negatively affects morphological growth and fruit development in melon (*Cucumis melo* L.). Chilling stress at the seedling stage causes seedling injury and poor stand establishment, prolonging vegetative growth and delaying fruit harvest. In this study, association mapping was performed for chilling tolerance at the seedling stage on an expanded melon core collection containing 212 diverse accessions by 272 SSRs and 27 CAPSs. Chilling tolerance of the melon seedlings was evaluated by calculating the chilling injury index (CII) in 2016 and 2017. Genetic diversity analysis of the whole accession panel presented two main groups, which corresponded to the two subspecies of *C. melo, melo*, and *agrestis*. Both the subspecies were sensitive to chilling but with *agrestis* being more tolerant. Genome-wide association study (GWAS) was conducted, respectively, on the whole panel and the two subspecies, totally detecting 51 loci that contributed to 74 marker-trait associations. Of these associations, 35 were detected in the whole panel, 21 in *melo*, and 18 in *agrestis*. About half of the associations identified in the two subspecies were also observed in the whole panel, and seven associations were shared by both the subspecies. CMCT505_Chr.1 was repeatedly detected in different populations with high phenotypic contribution and could be a key locus controlling chilling tolerance in *C. melo*. Nine loci were selected for evaluation of the phenotypic effects related to their alleles, which identified 11 elite alleles contributing to seedling chilling tolerance. Four such alleles existed in both the subspecies and six in either of the two subspecies. Analysis of 20 parental combinations for their allelic status and phenotypic values showed that the elite alleles collectively contributed to enhancement of the chilling tolerance. Tagging the loci responsible for chilling tolerance may simultaneously favor dissecting the complex adaptability traits and elevate the efficiency to improve chilling tolerance using marker-assisted selection in melon.

## Introduction

Melon (*Cucumis melo* L.), an important horticultural crop, is planted widely in both tropical and temperate regions around the world. This crop has abundant genetic diversity, especially for the fruit characters. Based on the morphological variation, melon cultivars are divided into two major varietal groups in China, thick-skinned and thin-skinned groups, corresponding, respectively, to the two subspecies, *melo* and *agrestis* (Pitrat, 2008). In China, recent years have seen an increasing plantation area of melon, resulting from the sustained demand for the multipurpose fruits in domestic market (Wang and Wu, 2014). Today, China has been the leading melon producer in the world with a fruit yield of over 13 million tons per year in the past decade, which accounts for nearly half of the world’s total yield (FAO Statistics from 2007 to 2016^1^). Production of melon in China is mainly observed as the protected cultivation that can put forward the harvest time by 1–2 months (Jiao et al., 2002), and obviously increases the income of the farmers. However, melon is a chilling-sensitive plant and often suffers from chilling injury that frequently occurs in early spring when melon plants are at seedling stage (Paull, 1990). Melon seedlings are more sensitive to low temperature than at other growth stages; in general, the seedlings stop growth at 10°C–13°C and display injury symptoms (e.g., leaf rolling, necrosis, and chlorosis) when the temperature falls below 8°C (Lin et al., 1995). This leads to prolongation of the seedling stage delaying fruit harvest and listing. Hence, promotion of the benefit of melon plantation is restricted by the cool temperatures occurring at the seedling stage. Developing new cultivars with enhanced chilling tolerance is helpful to solve this problem and has been a major focus in melon breeding programs (Luan et al., 2016).

Several studies have identified some chilling-tolerance materials in melon (Sun et al., 2004; Gao et al., 2016; Zhou et al., 2017); *agrestis* accessions are usually found to be more tolerant than *melo* accessions. Chinese historical literatures show that the early *agrestis* cultivars were usually planted under cool-temperature conditions, such as early spring and high elevations (e.g., Qimin Yaoshu, an agricultural literature of Northern Wei Dynasty). Due to mutual pollination between the two subspecies, some *melo* accessions or intermediate phenotypes acquire the chilling-tolerance genes. Identifying these materials with desirable genes is an essential step toward developing new cultivars with enhanced tolerance and promoting melon production in the cool-climate seasons.

Identification of the genes or QTLs related to low-temperature tolerance has been mainly focused on several model plants, such as *Arabidopsis* (Horton et al., 2016), rice (Zhang et al., 2014, 2018), maize (Huang et al., 2013), and tomato (Goodstal et al., 2005; Zhao et al., 2015). For these plants, almost each of the chromosomes bears the genes or QTLs conferring low-temperature tolerance at vegetative and reproductive stages, indicating the complex genetic basis of this trait. The genes isolated from the model plants may be divided into two categories: those that code the functional proteins protecting their cell and plasma membranes (e.g., AFPs), and the ones that code transcription factors (e.g., CBFs) or protein kinases (e.g., OsMAPK3) which regulate the downstream cold-response genes (Thomashow, 2010; Shi et al., 2015). Most of the genes were obtained by map-based cloning. No information is available on identification of the genes/QTLs related to low-temperature tolerance in melon to date.

Genome-wide association analysis (GWAS) is a powerful approach for identifying the target QTLs at the whole-genome level, which uses natural collections, cultivars or accessions (Hall et al., 2010). This approach has been applied to explore the QTLs related to chilling tolerance in several plant species, such as *Arabidopsis* (Horton et al., 2016), maize (Yan et al., 2011; Revilla et al., 2016), rice (Lv et al., 2016; Schläppi et al., 2017; Zhang et al., 2018), and sorghum (Fiedler et al., 2016; Parralondono et al., 2018). By means of high throughput genotyping, several of the studies mapped the QTLs at a high accuracy that involved a few to dozens of candidate genes (Lv et al., 2016; Revilla et al., 2016; Zhang et al., 2017; Parralondono et al., 2018). These facts demonstrate a power for this approach in screening of the chilling-tolerant QTLs in a range of plants, even the target genes. Today, GWAS has been successfully used in melon for identifying the QTLs affecting fruit firmness (Nimmakayala et al., 2016), fruit shape (Gur et al., 2017), soluble solids content (Xu et al., 2017), and other traits (Tomason et al., 2013; Wang et al., 2017).

Previously, we constructed a core collection from the National Mid-term Genebank for Watermelon and Melon (NMGWM) that contained over 1,200 melon accessions and coved 21 countries and regions in the world (Hu et al., 2013). Then, the primary core set (189 diverse accessions) was expanded by supplementing 23 chilling-tolerant accessions from USDA melon germplasm collection. The present study used this expanded core collection as natural population for GWAS, and discovered the associations between markers and chilling tolerance at melon seedling stage as well as the elite alleles in the accession panel. The results will provide the robust markers showing strong phenotypic effects and the potential donor parents for development of new cultivars with high chilling tolerance by molecular marker-assisted selection.

## Materials and Methods

### Plant Materials

Totally, 212 melon accessions were used for association mapping, which contained 189 diverse accessions (the core set constructed from NMGWM) and 23 accessions from USDA melon germplasm collection, and coved the major areas of origin of melons in the world (Supplementary Table S1). Among them, 135 belonged to subsp. *melo* and 77 to subsp. *agrestis* according to the classification suggested by Pitrat (2008). All the accessions have grown for more than five generations (in every spring from 2010 to 2015) in the greenhouse at the experimental farm of Henan Agricultural University (Scientific and Educational Park of Maozhuang, Zhengzhou). In our planting scheme, each accession containing 15–20 individuals was cultivated in a row every year and the self-fertilized seeds were harvested from the middle plants in each line.

In addition, 20 hybrids were prepared with their parents chosen from the melon panel based on identification of the alleles related to chilling tolerance in the next association mapping, and were used in the present study to examine the combination of potential elite alleles.

### Evaluation of Chilling Tolerance at the Seeding Stage

Chilling tolerance was assessed for all the accessions and hybrids following the procedure reported by our research group (Zhou et al., 2017). Briefly, melon seeds were immersed in 26°C–30°C water for 4 h and then transferred to a constant-temperature condition of 30°C for germination. When the seeds showed 1–3 mm radicles, they were sown in multi-plug trays (60 cells per tray) with each cell for one seed kernel. Each cell of the trays had a surface of 4 cm × 4 cm and a depth of 3.5 cm filled with commercial nursery substrates. Twenty seeds per accession were planted in two rows in a tray and thus each tray contained 60 seeds that belonged to three melon accessions. After that, the trays were placed in growth chambers (RLD-1000D-4, Le electron Co. Ltd., Ningbo, China) for seedling nursing, with a constant temperature of 25°C and a photoperiod of 16-h light/8-h dark. Until two-leaf stage, the weak seedlings were removed and healthy two-leaf seedlings with phenotypic uniformity (10–15 individuals per accession) were exposed to low temperature in a cold chamber with a setting of 75% relative humidity, a photoperiod of 16-h light/8-dark, and a light density of 360 μ mol s^-1^ m^-2^. This cold chamber (∼20 m^2^ in area) was built inside a lab with double-layer partitions, insulated with injected polyurethane. The low temperature treatment is to keep the seedlings at 25°C for 3 days, then gradually low the temperature from 25°C to 4°C with a gradient of 2°C/h, and finally maintained at 4°C for 48 h. Once the cold treatment was finished, phenotypic investigation was performed. Ten individuals per accession were randomly selected from the low temperature-treated seedlings and measured for their injury classes. Since the main environmental factors (temperature, humidity, and light density) were well controlled in the artificial cold chamber and showed no obvious difference over the spatial distribution, a complete randomized design was conducted on the experiment with three replications. Thus, each melon accession had 30 individuals in total for phenotypic record and the average of three replications was used for GWAS. Given that the experiment involved a large number of materials, phenotypic investigation was to photograph the cold-treated seedlings and then measure their injury classes via examination of the seedling images. That ensured the accuracy of the phenotypic data. The stress experiment was carried out in March 2016 and August 2017, respectively.

The phenotypic data of chilling tolerance were recorded by calculating the chilling injury index (CII) according to the published method (Semeniuk et al., 1986) but with some modification. Firstly, the low temperature-treated seedlings were determined for their injury classes as the followings:

Class 0: The seedlings are normal with no obvious injury symptom;

Class 1: The margin of real leaves curl and shrink slightly but with no hygrophanous spot;

Class 2: The margin of real leaves curl and shrink with small hygrophanous spots;

Class 3: The margin of real leaves curl and shrink with the area of hygrophanous spots accounting for about half of the leaf area;

Class 4: The margin of real leaves curl and shrink heavily with the area of hygrophanous spots exceeding half of the leaf area;

Class 5: The leaves become necrotic and the whole plant appears to be chlorotic or dies.

Then, the CII was calculated as the formula: CII = (1 × *N*_1_ + 2 × *N*_2_ + 3 × *N*_3_ + 4 × *N*_4_ + 5 × *N*_5_)/(*n* × 5), where *N*_1_-*N*_5_ mean the number of the seedling responding to Classes 1–5, respectively, and *n* means the total number of the seedlings tested. For this method, a high CII value means a low-level tolerance to chilling stress.

### Marker Polymorphism and Genotyping

Leaf tissues were sampled from the 3-week-old plants of the melon accessions. Genomic DNA was extracted from the young leaf tissues using the CTAB method. A total of 299 markers, 272 microsatellites (SSRs) (Supplementary Table S2) and 27 cleaved amplified polymorphic sequences (CAPSs) (Supplementary Table S3), were selected for even distribution across the 12 melon chromosomes and used for genotyping. Of these markers, 140 SSRs were chosen from the report of Zhu et al. (2016), 86 SSRs from the consensus linkage map of melon (Diaz et al., 2011), 31 SSRs from the database CmMDb (Chaduvula et al., 2015), and the rest 15 SSRs and 27 CAPSs were newly developed in our research group using next-generation sequencing. The distance between the markers averaged 785 kb ranging from 0.3 to 3,017 kb, with approximately 60% of the distances between adjacent markers were less than 800 kb. SSR-PCR reaction system, amplification program, and gel electrophoresis of PCR products were as previously described (Zhu et al., 2016). CAPS amplification, enzyme digestion, and agarose gel electrophoresis of enzyme-digested products were same as the report of Amanullah et al. (2018).

### Genome-Wide Association Analysis

Population structure of the accession panel as well as the *melo* and *agrestis* accessions was inferred using a model-based program STRUCTURE V2.3.4 (Pritchard et al., 2000) with 60 unlinked SSR markers from the 272 SSRs. For this analysis, the length of burn-in period was set at 10,000 followed by 100,000 Markov Chain Monte Carlo (MCMC) replications, with an admixture and allele frequency correlated model. Each of the probable K (the presumable number of subpopulations) was run for 10 times with the value ranging from 1 to 10. The optimal K was determined by the log probability of data [Lnp(D)] from the output and the Evanno’s ΔK between successive *K*-values (Evanno et al., 2005). Also, the software MEGA6 (Tamura et al., 2013) was used to construct a neighbor-joining (NJ) tree using the Nei’s genetic distance between pairwise individuals (Nei, 1978), aiming to verify further the genetic structure of the panel.

TASSELV2.1 (Bradbury et al., 2007) was used to seek the associations between the markers and the measures of chilling tolerance of melon seedlings, CII values. For this analysis, two regression models, general and mixed linear models (GLM and MLM), were compared by construction of quantile–quantile (Q–Q) plots which showed the distributions of *P*-values at the different models in comparison to the expected null hypothesis distribution. The optimal model was adopted to analyze the association between the markers and CII for the whole accession panel as well as the *melo* and *agrestis* accessions separately. The population structure array (Q) output from STRUCTURE V2.3.4 was used to control the false discovery in association analysis. Trait-marker associations were determined by the *P*-values, with the *R*^2^ indicating the fraction of the total variation explained by the marker identified. Phenotypic effects of the marker alleles were accessed using a method of “null allele” proposed by Breseghello and Sorrells (2006).

## Results

### Phenotypic Screening of Chilling Tolerance of Melon Seedlings

**FIGURE 1:**
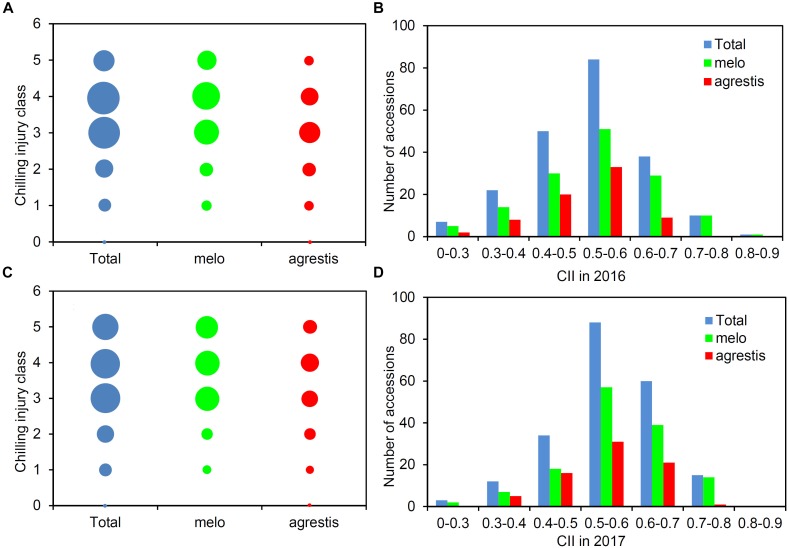
Phenotypic structure of the melon panel in response to low-temperature treatment. Distribution of the number of accessions with different chilling injury classes among the total accessions, *melo* accessions, and *agrestis* accessions in 2016 **(A)** and 2017 **(C)**. Distribution of the number of accessions with CIIs among the total accessions, *melo* accessions, and *agrestis* accessions in 2016 **(B)** and 2017 **(D)**.

A total of 212 melon accessions were evaluated for chilling tolerance at two-leaf stage. The chilling injury classes of these accessions were recorded and then used to calculate CIIs (Supplementary Table S1). Figure 1 presented the distribution of the chilling injury classes and CIIs for the whole panel as well as the two subspecies groups, displaying a wide range of variation among the groups. Largely, the classes were concentrated around 3–4 (Figures 1A,C), and the CII values were focused on 0.5–0.6 (Figures 1B,D). About 50% of the accessions were tolerant (classes 0–3) and the rest 50% were sensitive (classes 4–5). The CII values of the panel varied from 0.208 to 0.854, averaging 0.516 in 2016 and 0.551 in 2017. A low level of CII values (≤0.5) was observed with 95 accessions (44.81%) in 2016 and 58 (27.36%) in 2017.

As for the subspecies, nearly half of *agrestis* accessions (53.25% in 2016 and 45.45% in 2017) had a low class (≤3), while the percentage of *melo* accessions with class ≤ 3 was comparatively lower, with 42.22% in 2016 and 34.07% in 2017. The CII values of *agrestis* accessions varied from 0.208 to 0.750 in 2016 (mean = 0.483) and 0.281 to 0.750 in 2017 (mean = 0.511), and those of *melo* accessions from 0.220 to 0.854 in 2016 (mean = 0.534) and 0.250 to 0.751 in 2017 (mean = 0.563) (Table 1). These findings indicated that *agrestis* accessions had a higher chilling tolerance than *melo* accessions.

**Table 1:** Phenotypic variation for chilling tolerance of the total accessions and the two subspecies.

Accessions	Year	Mean ± SD^a^	Range	CV^b^ (%)	Kurtosis	Skewness	H^2^_B_^c^ (%)
The whole panel	2016	0.516 ± 0.114	0.208–0.854	22.13	-0.044	-0.150	93.13
	2017	0.551 ± 0.099	0.250–0.750	18.04	0.182	-0.463	89.69
*melo*	2016	0.534 ± 0.115	0.220–0.854	21.50	-0.189	-0.025	91.46
	2017	0.563 ± 0.103	0.250–0.750	18.25	0.229	-0.526	87.32
*agrestis*	2016	0.483 ± 0.106	0.208–0.650	21.99	-0.364	-0.622	88.45
	2017	0.531 ± 0.091	0.281–0.750	17.05	0.265	-0.548	86.07


*^*a*^SD, standard deviation; ^*b*^CV, coefficient of variation; ^*c*^H^*2*^_B_, heritability in the broad sense.*

The phenotypic segregation of CII values was close to fitting a normal distribution with a slight negative skewness, as indicated by the Kurtosis and Skewness values (Table 1). Therefore, the CII values of the whole panel or subspecies groups were suitable for association mapping.

### Genetic Diversity and Population Structure of the Panel

All the 299 markers (272 SSRs and 27 CAPSs) revealed a genome-wide distribution and a high-level polymorphism. A total of 2,339 alleles were detected, with a mean allelic number per locus of 7.82 varying from 2 to 15. As a measurement of the genetic diversity, polymorphic information content (PIC) varied from 0.148 for SSR015119 to 0.889 for HSSR010, with an average of 0.573. Two hundred and thirty-three markers (77.93%) showed high polymorphism (PIC > 0.5) and the rest 66 markers (22.07%) were less informative (PIC < 0.5). SSRs were much more informative than CAPSs in that over half of CAPSs showed low PIC values (PIC < 0.5).

**FIGURE 2:**
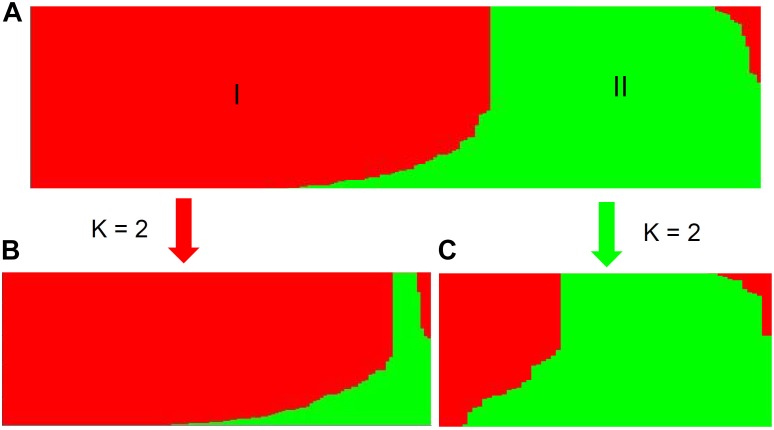
Population structure of the whole accession panel and the two subspecies inferred from STRUCTURE analysis. The whole panel **(A)** was divided into two main groups (I and II). Each of the two subspecies, *melo* accessions **(B)** and *agrestis* accessions **(C)**, was further subdivided into two subgroups, where *K* = 2.

**FIGURE 3:**
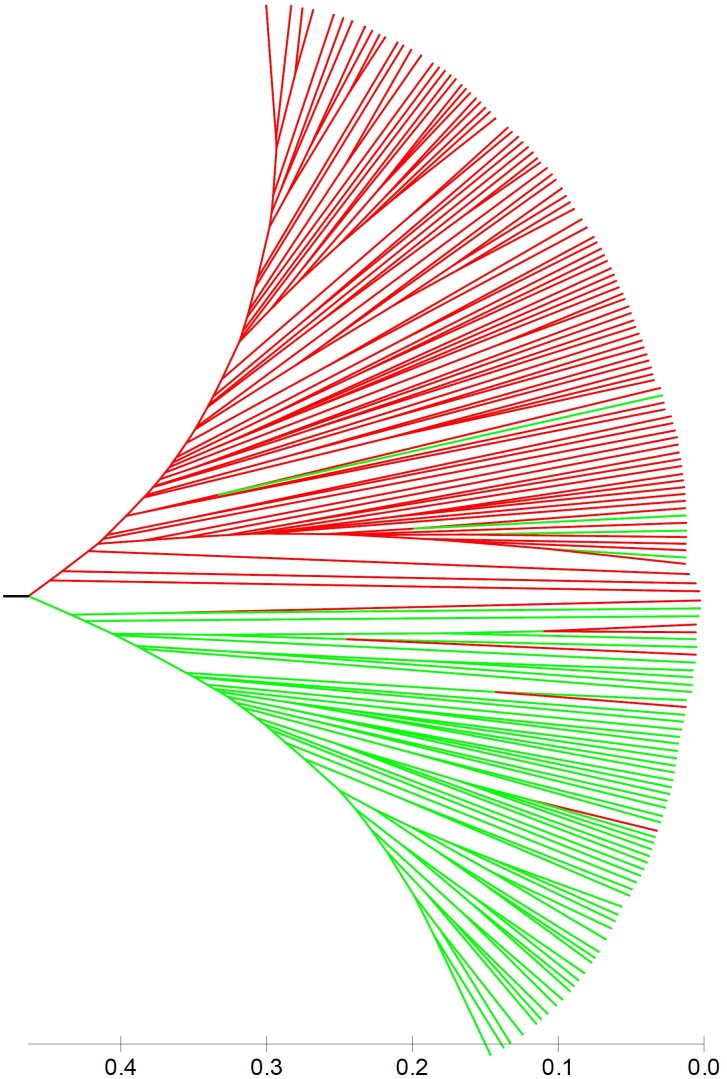
A neighbor-joining tree of 212 melon accessions showing two main clusters. Red and green lines represented *melo* accessions and *agrestis* accessions, respectively.

Population stratification is one of the major causes of confounding in association analysis, and hence, genetic structure existed in natural population needs to be determined to avoid false-positive associations for GWAS. In the STRUCTURE program, the posterior probability Lnp(D) progressively increased with the increase of the K values, leading to indetermination of the true value of K. So the Evanno’s ΔK was calculated for all K classes which showed a strong peak at *K* = 2 (Supplementary Figure S1A). Accordingly, the 212 melon accessions could be divided into two main groups (I and II) (Figure 2), largely corresponding to the two subspecies, *melo* and *agrestis*. Group I contained 131 *melo* accessions and 4 *agrestis* accessions, while group II consisted of 71 *agrestis* accessions and 6 *melo* accessions. Also, each of the two subspecies groups could be further subdivided to two subgroups, where the ΔK values gave *K* = 2 to both groups I and II (Supplementary Figures S1B,C). Alternatively, a neighbor-joining (NJ) tree based on the proportion of shared alleles coefficient was constructed with the two distinct clusters (green and red regions) (the bootstrap value > 60%), demonstrating that the whole accession panel was clustered into two major clades (Figure 3). Clearly, the clustering results were similar to those obtained using STRUCTURE, while each of the two clusters could not be subdivided.

### Mapping of the Loci Underlying Chilling Tolerance

**FIGURE 4:**
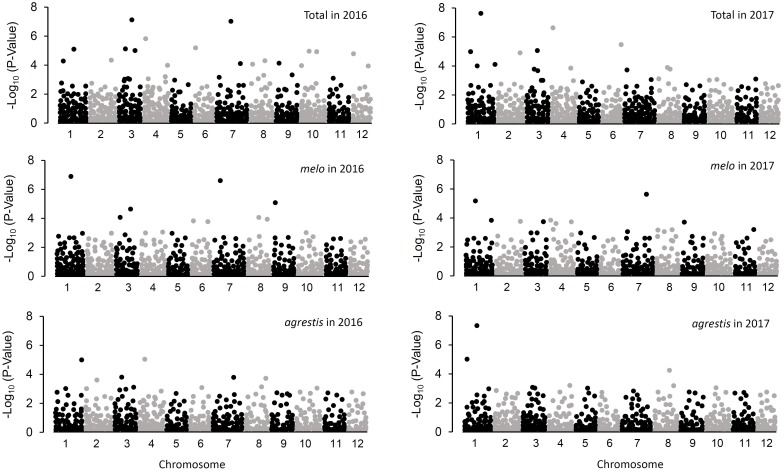
Manhattan plots resulting from GWAS for CII values of melon seedlings.

Comparison of GLM and MLM models for GWAS was performed by construction of Q–Q plots (Supplementary Figure S2), which showed that MLM underestimated the associations between genotype and phenotype. Therefore, GLM was more suitable for GWAS in the whole collection as well as the two subspecies groups. The respective Q matrix outputs of the whole panel and the two subspecies groups from STRUCTURE analysis were used as covariates in the association analysis. In total, 74 trait-marker associations involving 51 marker loci were detected at the level of probability of *P* < 0.005, of which 35 associations were observed in the whole panel, 21 in *melo* group, and 18 in *agrestis* group (Figure 4 and Table 2). Except for two associations with CAPSs (M34P and M8-1P), all the rest 72 associations were detected with SSR markers, indicating a higher efficiency of SSR markers in GWAS.

**Table 2:** Summary of the marker loci significantly associated with CII values mapped in the whole panel and the two subgroups.

Population	Locus	Chr.	Position (Mb)	2016		2017	
					
				*R*^2a^	*P*-value	*R*^2^	*P*-value
The whole panel	SSR009175	1	6.03	0.118	6.56E-04	0.152	3.70E-05
	SSR009461	1	10.99	–^b^	ns^c^	0.074	1.90E-04
	CMCT505	1	16.53	0.204	2.43E-05	0.166	5.10E-08
	SSR010787	1	25.77	–	ns	0.133	1.12E-04
	DE1329	2	23.83	0.214	5.56E-04	0.107	3.02E-05
	M34P	3	9.53	0.070	7.55E-05	–	ns
	SSR015399	3	10.02	0.098	1.12E-03	0.131	1.40E-04
	SSR015533	3	12.12	0.053	3.92E-03	0.099	3.87E-05
	SSR015603	3	13.55	–	ns	0.092	4.14E-04
	SSR015784	3	16.68	0.187	3.38E-07	0.126	4.01E-03
	SSR015829	3	17.05	0.208	4.22E-05	0.115	3.05E-03
	CMTAN142	4	3.95	0.342	5.90E-06	0.191	7.23E-07
	SSR018207	4	6.20	0.104	2.20E-03	–	ns
	SSR019550	4	22.24	0.089	1.04E-03	0.210	2.77E-04
	SSR019803	4	24.14	0.068	8.52E-04	0.106	3.41E-03
	DE1035	5	1.07	0.100	3.59E-03	0.037	4.11E-03
	HNM41	6	4.57	0.211	6.18E-05	–	ns
	CMCTN38	6	35.85	–	ns	0.082	2.16E-05
	CMAGN75	7	2.40	0.086	2.34E-03	0.114	1.24E-04
	SSR028465	7	17.42	0.117	6.78E-07	–	ns
	ECM182	7	21.01	0.144	2.29E-04	0.103	1.20E-03
	ECM88	8	1.15	–	ns	0.098	1.05E-03
	HNM2	8	1.80	0.130	3.23E-04	–	ns
	M8-1P	8	7.82	0.077	3.06E-03	–	ns
	HNM31	8	11.15	0.050	2.50E-03	0.111	2.24E-04
	SSR031575	8	12.20	0.226	1.05E-04	0.127	5.67E-04
	SSR032562	9	0.17	0.100	6.22E-04	–	ns
	SSR034244	9	18.01	0.049	3.50E-03	–	ns
	SSR035466	10	1.33	0.069	4.20E-04	0.086	3.11E-03
	SSR036720	10	13.39	0.122	5.87E-05	0.097	2.77E-03
	HSSR002	10	20.80	0.207	5.02E-05	–	ns
	SSR036952	11	0.130	0.065	1.85E-03	–	ns
	SSR038777	11	21.61	–	ns	0.120	1.12E-03
	DE1917	12	2.66	0.150	9.47E-05	–	ns
	HNM38	12	23.84	0.054	7.02E-04	–	ns
*melo*	CMCT505	1	16.53	0.306	2.24E-07	0.140	1.01E-05
	SSR010993	1	28.03	0.197	7.74E-03	0.103	6.72E-04
	DE1329	2	23.83	0.208	2.30E-03	0.070	3.36E-04
	CSWCT10	3	3.92	0.075	6.72E-04	–	ns
	SSR015399	3	10.02	0.133	7.36E-03	0.225	4.03E-03
	SSR015829	3	17.05	0.223	2.24E-05	0.101	4.38E-03
	CMCTTN175	3	20.90	–	ns	0.141	2.24E-04
	SSR017422	4	0.27	–	ns	0.117	3.36E-04
	CMTAN142	4	3.95	–	ns	0.094	1.01E-03
	SSR018207	4	6.20	0.213	2.46E-03	0.078	6.72E-04
	SSR019550	4	22.24	0.096	4.39E-03	0.059	6.72E-04
	HNM41	6	4.57	0.169	6.12E-04	–	ns
	SSR026426	6	25.84	0.142	4.48E-04	–	ns
	CMAGN75	7	2.40	0.202	1.12E-07	0.093	5.39E-03
	ECM182	7	21.01	–	ns	0.247	2.32E-06
	ECM88	8	1.15	–	ns	0.079	4.48E-03
	SSR031575	8	12.20	0.197	1.57E-04	0.106	5.11E-03
	CMAT141	8	26.43	0.161	2.51E-04	0.050	3.33E-03
	SSR032562	9	0.17	0.069	8.96E-05	0.075	8.76E-04
	SSR036581	10	11.21	0.048	3.01E-03	–	ns
	SSR038830	11	22.07	–	ns	0.120	1.91E-03
*agrestis*	HNM10	1	2.81	–	ns	0.103	8.95E-05
	CMCT505	1	16.53	0.147	5.22E-03	0.221	4.10E-07
	SSR010993	1	28.03	0.195	4.07E-05	0.088	1.01E-03
	HNM15	2	0.18	0.063	5.30E-03	0.121	3.92E-03
	TJ24	2	15.39	0.071	2.46E-04	–	ns
	SSR014992	3	4.79	0.101	3.92E-03	–	ns
	SSR015399	3	10.02	0.077	8.92E-04	0.084	4.59E-03
	SSR015829	3	17.05	0.119	5.26E-03	0.122	4.61E-03
	CMCTTN175	3	20.90	0.090	2.13E-03	–	ns
	CMTAN142	4	3.95	0.114	4.48E-05	–	ns
	SSR019803	4	24.14	–	ns	0.068	2.80E-03
	SSR022483	5	17.73	–	ns	0.048	3.58E-03
	SSR025828	6	18.79	0.086	2.13E-03	–	ns
	ECM182	7	21.01	0.133	3.25E-04	–	ns
	HNAM40	8	20.88	0.106	3.25E-03	0.202	3.38E-04
	SSR032536	8	24.10	0.192	6.07E-04	0.116	4.03E-03
	SSR036720	10	13.39	–	ns	0.080	5.34E-03
	HSSR002	10	20.80	0.105	3.70E-03	–	ns


*^*a*^*R*^*2*^ the proportion of the total phenotypic variance explained by the SSR locus; ^*b*^not given; ^*c*^ns, no significant.*

As for the whole panel, the 35 loci were distributed on all the 12 chromosomes, with the maximum number of 6 on Chr.3 and only one on Chr.2 and Chr.5. These loci explained a small proportion of the total phenotypic variance (*R*^2^), varying from 0.037 (DE1035_Chr.5) to 0.342 (CMTAN142_Chr.4) (mean = 0.122). Eight loci, i.e., CMCT505_Chr.1, DE1329_Chr.2, SSR015829_Chr.3, CMTAN142_Chr.4, SSR019550_Chr.4, HNM41_Chr.6, SSR031575_Chr.8, and HSSR002_Chr.10, showed a high level of *R*^2^ value (>0.2), suggesting an existence of major QTLs around these loci affecting chilling tolerance of melon seedlings. About half of the loci were detected repeatedly in both 2016 and 2017. Interestingly, a big QTL could be located on Chr.3 which covered a distance span of 9.53–17.05 Mb, as indicated by the six successive markers in this region that associated with CIIs.

In *melo* group, 21 associations were detected on 10 chromosomes; the maximum number of 4 occurred on Chr.3 while no association on Chr.5 and Chr.12. Eleven of these loci were repeatedly detected in the two environments. The *R*^2^ values ranged from 0.050 (CMAT141_Chr.8) to 0.306 (CMCT505_Chr.1) with a mean of 0.134. Six loci (CMCT505_Chr.1, DE1329_Chr.2, SSR015829_Chr.3, SSR015399_Chr.3, SSR018207_Chr.4, CMAGN75_Chr.7) had a comparatively high phenotypic contribution with *R*^2^ > 0.2.

Eighteen associations were detected in *agrestis* group distributing across nine chromosomes, with the maximum number of four on Chr.3 and no association on Chr.9, Chr.11, and Chr.12. Compared to those detected in *melo*, these loci contributed to a lower proportion of phenotypic variance in that only two loci (CMCT505_Chr.1 and HNAM40_Chr.8) had a *R*^2^ value of >0.2, and the others’ values were lower than 0.2. The *R*^2^ values of the 18 loci averaged 0.114 varying from 0.048 (SSR022483_Chr.5) to 0.221 (CMCT505_Chr.1). Seven loci appeared repeatedly in the two consecutive years.

Comparison of the associations detected in different groups presented some common or specific loci (Table 2). Fourteen loci in *melo* group were also observed in the whole panel, whereas 9 in *agrestis* group appeared in the whole panel. Seven loci, i.e., CMCT505_Chr.1, SSR015399_Chr.3, SSR015829_Chr.3, CMCTTN175_Chr.3, CMTAN142_Chr.4, ECM182_Chr.7, and ECM88_Chr.8, were shared by both the subspecies groups. Also, there were several specific loci that belonged to a certain subspecies; seven were specific to *melo* and eight to *agrestis*. Of these loci, what deserves attention more was CMCT505_Chr.1 because of its stable detection and high phenotypic contributions. The candidate genes linked genomic region of CMCT505_Chr.1 were chosen based on the genomic sequence of the melon genotype DHL92 (Garcia-Mas et al., 2012) with an interval of 100-kb region flanking upstream and downstream from this locus. We identified 15 candidate genes for this locus probably associated with chilling tolerance, as summarized in Supplementary Table S4.

### Exploration of the Elite Alleles Conferring Chilling Tolerance

Since CII represented the degree of chilling injury of melon seedlings, the marker alleles with obviously negative effects were considered as elite alleles. For exploitation of elite alleles, nine loci with high *R*^2^ values (more than the means) and stable detection in different environments were selected, and the phenotypic effects of the different alleles of these loci were evaluated using the method of Breseghello and Sorrells (2006), as shown in Table 3. Totally, 27 alleles with distinct amplification products, which were designated by their band sizes, were identified for the selected loci. Of these, 11 alleles generated remarkably negative effects leading to enhancement of seedling chilling tolerance, and hence, were deemed as elite alleles. Four alleles produced negative effects in both the subspecies, such as CMCT505_160 bp, SSR015399_273 bp, SSR015829_205 bp, and SSR015829_214 bp. Four alleles (SSR010993_210 bp, DE1329_209 bp, SSR018207_270 bp, and SSR031575_195 bp) exerted negative effects in *melo* and two alleles (SSR015399_270 bp and HNAM40_205 bp) had the same function in *agrestis*. The remained one (CMCT505_203 bp) produced negative effects in *melo* but positive effects in *agrestis*.

**Table 3:** Phenotypic effects of the marker alleles significantly associated with chilling tolerance.

Locus	Allele (bp)	*melo*		*agrestis*	
			
		2016	2017	2016	2017
CMCT505	160	-13.17	-3.48	-15.56	-3.07
	182	2.84	2.94	–^a^	–
	203	-21.51	-9.84	2.54	6.33
	205	5.22	2.91	-1.24	3.07
SSR010993	208	-0.906	1.04	0.72	5.56
	210	-11.43	-5.59	–	–
	215	-2.43	1.50	4.05	2.27
	220	3.93	5.70	-0.67	-5.08
DE1329	200	1.17	-2.45	–	–
	209	-15.33	-6.93	–	–
	213	4.32	7.75	–	–
SSR015399	270	–	–	-8.93	-16.67
	273	-2.63	-2.69	-15.02	-8.34
	280	8.06	1.34	0.89	2.77
SSR015829	205	-4.33	-8.73	-19.44	-7.06
	214	-10.14	-7.87	-6.63	-8.49
	217	3.80	6.49	–	–
	220	12.44	5.88	6.03	2.85
SSR018207	261	2.64	5.47	–	–
	270	-5.44	-11.90	–	–
SSR031575	189	7.10	2.55	–	–
	195	-8.91	-19.79	–	–
HNAM40	200	–	–	0.919	6.77
	205	–	–	-6.05	-20.80
SSR032536	205	–	–	10.42	6.06
	209	–	–	-3.90	-2.83
	214	–	–	19.32	7.66


*^*a*^not detected.*

In order to assess the efficiency of these elite alleles, 20 parental combinations were prepared and evaluated for their phenotypic values, the CII values at the seedling phase. The hybrids and their parents of the cross combinations were examined by PCR for existence of the elite alleles detected above or not. Table 4 characterized the 20 hybrids for the number of elite alleles and phenotypic values in two environments. Five combinations (No. 2, 6, 9, 14, and 19) bore at least five elite alleles and exhibited a high level of chilling tolerance with the CII values being lower than 0.3. In contrast, seven combinations with a small number of elite alleles (<4) (No. 3, 8, 10, 12, 13, 16, and 18) were sensitive to chilling with the CII values of >0.5. This indicated that the elite alleles collectively contributed to enhancement of the chilling tolerance in an individual plant. However, there are some exceptions, e.g., Yinhui × PI234607 (No. 7) carried six elite alleles but was sensitive to chilling (the CII values more than 0.5 in both years), and Liu9602 × Bailangua (No. 15) lacked elite allele but showed a medium level of tolerance (the 2-year CII values around 0.4). Furtherly, the correlation was examined between the number of elite alleles and the corresponding CII values, with the correlation coefficients of -0.801 in 2016 (*P* < 0.01) and -0.781 in 2017 (*P* < 0.01).

**Table 4:** Phenotypic values and number of elite alleles of the 20 cross combinations.

No.	Parental combinations	Number of elite alleles	Phenotypic values	
			
			2016	2017
1	PI169329 × Yinhui	5	0.302	0.337
2	PI169329 × Bajiang-2	6	0.256	0.300
3	Ames29858 × Bailangua	2	0.605	0.630
4	Ames29858 × Yinhui	5	0.314	0.309
5	PI381765 × Zhalaapan	4	0.380	0.411
6	PI381765 × PI234607	5	0.267	0.225
7	Yinhui × PI234607	6	0.511	0.565
8	Yinhui × Xinshiji	3	0.555	0.527
9	Yinhui × Bajiang-2	7	0.190	0.209
10	Furong × Bailangua	1	0.705	0.683
11	Furong × Zhalaapan	2	0.495	0.468
12	PI234607 × Bailangua	3	0.567	0.550
13	PI166190 × Bailangua	2	0.598	0.622
14	PI166190 × Bajiang-2	6	0.217	0.203
15	Liu9602 × Bailangua	0	0.445	0.471
16	Liu9602 × Zhalaapan	1	0.610	0.632
17	Honeylew × PI508450	6	0.357	0.332
18	Honeylew × Fenghuang	2	0.693	0.701
19	Shushugua × PI508450	7	0.186	0.212
20	Liu9602 × PI508450	4	0.565	0.571


## Discussion

Low temperature rigidly limits the geographic distribution of thermophilic plants and influences fruit growth and maturity of these crops (Crawford, 2008). Melon is such a fruit plant with chilling sensitivity, and commonly suffers from chilling injury. Wild melons are believed to be highly resistant to chilling (Pitrat, 2013) but they are not easy to be applied in breeding practice as linkage drag often occurs during trait transfer. In such a case, core collection offers an opportunity for seeking the chilling-tolerance genes/QTLs as it comprised diverse accessions as well as those materials with chilling tolerance. The natural population for association mapping in the present study was actually an expanded core collection containing a number of such chilling-tolerance accessions, as demonstrated by the CII values in two consecutive years (Supplementary Table S1). It was clear that this collection enriched the genes related to chilling tolerance and favored elite gene discovery. Successful utilization of core collection for marker-trait association has been reported in a variety of plant species (Zhao et al., 2010; Li et al., 2011; Jiang et al., 2014).

There existed distinct population structures in the accession collection used in our study, as two main groups (I and II) corresponding to the two subspecies, *melo* and *agrestis*, were observed using two different methods. The similar results were also reported in other studies (Luan et al., 2008; Hu et al., 2014). This could imply a basic differentiation in *C. melo*, inter-subspecific differentiation. The two subspecies revealed different CII values with *agrestis* being more tolerant (Table 1), which is in accord with the available reports (Lin et al., 1995; Gao et al., 2016) and the practical plantation patterns in China. It is generally believed that *agrestis* was intensively domesticated in Chinese Central Plains (Kerje and Grum, 2000; Lin, 2010), where the climate diversity could endow this type of accessions with the tolerance to diverse stresses including low temperature.

Despite the existence of genetic divergence, the two subspecies had several same loci (CMCT505_Chr.1, SSR015399_Chr.3, SSR015829_Chr.3, CMCTTN175_Chr.3, CMTAN142_Chr.4, ECM182_Chr.7, and ECM88_Chr.8) that influenced chilling tolerance of melon seedlings. These shared loci could be derived from the gene exchange between the two subspecies, e.g., the mutual pollination. Since these loci were detected in various groups, they represented the chilling tolerance-related loci prevailing in *C. melo* and could be used for improvement of *C. melo* chilling tolerance. Certainly, most of the loci were specific to a certain subspecies, perhaps resulting from the specific domestication process. These subspecies-specific loci also play role in enhancing the chilling tolerance of the corresponding subspecies cultivars. It should be noted that the locus CMCT505_Chr.1 was stably detected in the two groups with high contributions to phenotypic variance. Clearly, it is probably a key locus controlling chilling tolerance in *C. melo*, and around this locus, there may be exist a major QTL that will help to discern the genetic control of chilling tolerance if fine mapping of this region is performed. Also, this locus has been reported to be located in the resistance-gene homologs of the melon genome (Brotman et al., 2000) and closely linked to gummy stem blight-resistance gene (*Gsb-1*) in melon (Bi et al., 2015). In cucumber, this locus was found to be linked to heat tolerance (Yang, 2007). Therefore, CMCT505 is likely to involve in the tolerance to diverse stresses, which further expands its potential value in cucurbit breeding. Up to now, no report was available on QTL mapping for chilling tolerance in melon yet, and only a few low-temperature-responsive genes were isolated by homology-based cloning (Zhang et al., 2017). However, a number of such genes have been cloned in model plants (Thomashow, 2010; Zhang et al., 2014; Shi et al., 2015; Zhao et al., 2015), and most of them involved in CBF-dependent cold signaling pathway. This offered an important reference for pursuing the chilling tolerance-related genes in the candidate regions of melon genome.

An association locus generally includes several alleles, some of which exert phenotypic effects to improve the target traits and are considered as elite alleles. In our study, such 11 elite alleles exerted strong negative effects leading to low CII values. Some of these alleles (CMCT505_160 bp, SSR015399_273 bp, SSR015829_205 bp, and SSR015829_214 bp) played a stable function in the two subspecies, and could act as the primary tolerant genes extensively existing in *C. melo*. The other elite alleles were subspecies-specific and might be resulted from the intra-subspecific domestication. These elite alleles deserved to positive selection in MAS-based chilling tolerance improvement. CMCT505_203 bp was an exception that it produced negative effects in *melo* but positive effects in *agrestis*. Some bi-parental crosses with enhanced chilling tolerance (No. 1, 2, 4, and 6) carried this allele but it was also observed in chilling-sensitivity crosses (No. 7, 8, and 12). Perhaps, this allele derived from the mutation of a certain sensitive gene in *melo*, and was used specially for tolerance improvement in *melo* cultivars. A comprehensive analysis of allelic components of the 20 bi-parental crosses showed that pyramiding the elite alleles into an individual plant led to enhancement of chilling tolerance in hybrids (Table 4), as further confirmed by the correlation analysis result that the number of elite alleles in an individual plant determined its degree of chilling tolerance. Several available reports also documented the similar findings that multiple elite alleles collectively contributed to high level of phenotypic effects (Jiang et al., 2013; Zhang et al., 2013; Cai et al., 2014). These findings not only offered a strategy for improvement of melon chilling tolerance (i.e., polymerization breeding), but also predicted the degree of chilling tolerance of a certain bi-parental cross through examination of the progeny allelic components. Nevertheless, not all the cross progenies carrying elite alleles showed tolerance enhancement, such as Yinhui × PI234607. Probably, some other inferior alleles, which were not detected in the present study, existed in these cross progenies. More accurate appraisal of phenotypic effects based on allelic variation needs further promotion of the marker density to identify more alleles involving in melon chilling tolerance.

## Author Contributions

J-BH planned and designed the experiments. Y-FZ, L-YG, Y-LW, C-SM, and S-RS carried out the experiments in growth chamber and field. Y-FZ, L-YG, H-YZ, and S-JZ carried out the molecular experiments. J-BH and L-MY analyzed the data and performed GWAS. JH and J-BH wrote the manuscript. J-BH and J-MW provided the genetic materials and revised the manuscript. All authors read and approved the final manuscript.

## Conflict of Interest Statement

The authors declare that the research was conducted in the absence of any commercial or financial relationships that could be construed as a potential conflict of interest. The reviewer FL declared a past co-authorship with one of the authors JH to the handling Editor.

## Footnotes

1 http://www.fao.org/faostat/en/#data/QC

## References

[B1] Amanullah S. Liu S. Gao P. Zhu Z. Zhu Q. Fan C. (2018). QTL mapping for melon (*Cucumis melo L*.) fruit traits by assembling and utilization of novel SNPs based CAPS markers. *Sci. Hortic.* 236 18–29. 10.1016/j.scienta.2018.02.041

[B2] Bi Y. Xu B. Qian C. Guo J. Zhang Y. Yin H. (2015). Pyramiding disease resistance genes and variety improvement by molecular marker-assisted selection in melon (*Cucumis melon L.*). *Sci. Agric. Sin.* 48 523–533. 10.3864/j.issn.0578-1752.2015.03.12

[B3] Bradbury P. Zhang Z. Kroon D. Casstevens T. Ramdoss Y. Buckler E. (2007). TASSEL: software for association mapping of complex traits in diverse samples. *Bioinformatics* 23:2633. 10.1007/s00122-011-1719-0 17586829

[B4] Breseghello F. Sorrells M. E. (2006). Association mapping of kernel size and milling quality in wheat (*Triticuma estivum L*.) cultivars. *Genetics* 172 1165–1177. 10.1534/genetics.105.044586 16079235 PMC1456215

[B5] Brotman Y. Silberstein L. Kovalski I. Klingler J. Thompson G. Katzir N. (2000). Linkage groups of *Cucumis melo*, including resistance gene homologues and known genes. *Acta Hortic.* 510 441–448. 10.17660/ActaHortic.2000.510.70

[B6] Cai C. Ye W. Zhang T. Guo W. (2014). Association analysis of fiber quality traits and exploration of elite alleles in upland cotton cultivars/accessions (*Gossypium hirsutum L.*) *J. Integr. Plant Biol.* 56 51–62. 10.1111/jipb.12124 24428209

[B7] Chaduvula B. P. K. Bonthala V. S. Manjusha V. Siddiq E. A. Polumetla A. K. Prasad G. M. (2015). CmMDb: a versatile database for *Cucumis melo* microsatellite markers and other horticulture crop research. *PLoS One* 10:e0118630. 10.1371/journal.pone.0118630 25885062 PMC4401682

[B8] Crawford R. M. M. (2008). *Plants at the Margin: Ecological Limits and Climate Change.* Cambridge: Cambridge University Press 10.1017/CBO9780511754906

[B9] Diaz A. Fergany M. Formisano G. Ziarolo P. Blanca J. Fei Z. (2011). A consensus linkage map for molecular markers and quantitative trait loci associated with economically important traits in melon (*Cucumis melo L.*). *BMC Plant Biol.* 11:111. 10.1186/1471-2229-11-111 21797998 PMC3163537

[B10] Evanno G. Regnaut S. Goudet J. (2005). Detecting the number of clusters of individuals using the software structure: a simulation study. *Mol. Ecol.* 14 2611–2620. 10.1111/j.1365-294X.2005.02553.x 15969739

[B11] Fiedler K. Bekel W. A. Matschegewski C. Snowdon R. Wieckhorst S. Zacharias A. (2016). Cold tolerance during juvenile development in sorghum: a comparative analysis by genome wide association and linkage mapping. *Plant Breed.* 135 598–606. 10.1111/pbr.12394

[B12] Gao Q. Wang Y. Guo Y. (2016). Identification for tolerance to low temperature and weak light and selection for morphological indices of melon at seedling stage. *Acta Agric.* 28 1360–1367. 10.3969/j.sssn.1004-1524.2016.08.13

[B13] Garcia-Mas J. Benjak A. Sanseverino W. Bourgeois M. Mir W. González V. M. (2012). The genome of melon (*Cucumis melo L.*). *Proc. Natl. Acad. Sci. U.S.A.* 109 11872–11877. 10.1073/pnas.1205415109 22753475 PMC3406823

[B14] Goodstal F. J. Kohler G. R. Randall L. B. Bloom A. J. St. Clair D. A. (2005). A major QTL introgressed from wild *Lycopersicon hirsutum* confers chilling tolerance to cultivated tomato (*Lycopersicon esculentum*). *Theor. Appl. Genet.* 111 898–905. 10.1007/s00122-005-0015-2 16075210

[B15] Gur A. Tzuri G. Meir A. Sa’ar U. Portnoy V. Katzir N. (2017). Genome-wide linkage-disequilibrium mapping to the candidate gene level in melon (*Cucumis melo*). *Sci. Rep.* 7:e9770. 10.1038/s41598-017-09987-4 28852011 PMC5575340

[B16] Hall D. Tegstrom C. Ingvarsson P. K. (2010). Using association mapping to dissect the genetic basis of complex traits in plants. *Breif Funct. Genomics* 9 157–165. 10.1093/bfgp/elp048 20053815

[B17] Horton M. W. Willems G. Sasaki E. Koornneef M. Nordborg M. (2016). The genetic architecture of freezing tolerance varies across the range of *Arabidopsis thaliana*. *Plant Cell Environ.* 39 2570–2579. 10.1111/pce.12812 27487257

[B18] Hu J. Ma S. Wang J. Su Y. Li Q. (2013). Establishment of a melon (*Cucumis melo*) core collection based on phenotypic characters. *J. Fruit Sci.* 30 404–411.

[B19] Hu J. Wang P. Su Y. Wang R. Li Q. Sun K. (2014). Microsatellite diversity, population structure, and core collection formation in melon germplasm. *Plant Mol. Biol. Rep.* 33 439–447. 10.1007/s11105-014-0757-6

[B20] Huang J. Zhang J. Li W. Hu W. Duan L. Feng Y. (2013). Genome-wide association analysis of ten chilling tolerance indices at the germination and seedling stages in maize. *J. Integr. Plant Biol.* 55 735–744. 10.1111/jipb.12051 23551400

[B21] Jiang H. Huang L. Ren X. Chen Y. Zhou X. Xia Y. (2014). Diversity characterization and association analysis of agronomic traits in a Chinese peanut (*Arachis hypogaea L*.) mini-core collection. *J. Integr. Plant Biol.* 56 159–169. 10.1111/jipb.12132 24237710

[B22] Jiang J. Chen L. Liu Q. He Y. Hong D. (2013). Mining elite alleles of growth duration and productive panicle number per plant by association mapping with conditional phenotypic value in Japonica rice. *Rice Sci.* 20 200–206. 10.1016/S1672-6308(13)60126-2

[B23] Jiao Z. G. Wang C. Q. Dong Y. M. Xiao S. H. Ma C. M. Li X. J. (2002). *Protected Cultivation of Watermelon and Melon.* Jinan: Shandong Science and Technology Press.

[B24] Kerje T. Grum M. (2000). The origin of melon, *Cucumis melo*: a review of the literature. *Acta Hortic.* 510 37–44. 10.17660/ActaHortic.2000.510.5

[B25] Li X. Yan W. Agrama H. Jia L. Shen X. Jackson A. (2011). Mapping QTLs for improving grain yield using the USDA rice mini-core collection. *Planta* 234 347–361. 10.1007/s00425-011-1405-0 21479810

[B26] Lin D. (2010). Origin, classification and evolution for cultivated plants of Chinese melon. *China Cucurbits Veg.* 23 34–36.

[B27] Lin D. Wu M. Wang J. (1995). *Cultivation of Melon for High Quality and Yield.* Beijing: Jindun Publishing House.

[B28] Luan F. Delannay I. Staub J. E. (2008). Chinese melon (*Cucumis melo L.*) diversity analyses provide strategies for germplasm curation, genetic improvement, and evidentiary support of domestication patterns. *Euphytica* 164 445–461. 10.1007/s10681-008-9699-0

[B29] Luan F. Wang X. Gao M. Gao P. Ma H. (2016). *Watermelon Melon Breeding and Biotechnology.* Beijing: Science Press, China.

[B30] Lv Y. Guo Z. Li X. Ye H. Li X. Xiong L. (2016). New insights into the genetic basis of natural chilling and cold shock tolerance in rice by genome-wide association analysis. *Plant Cell Environ.* 39 556–570. 10.1111/pce.12635 26381647

[B31] Nei M. (1978). Estimation of average heterozygosity and genetic distance from a small number of individuals. *Genetics* 89 583–590. 17248844 10.1093/genetics/89.3.583 PMC1213855

[B32] Nimmakayala P. Yan R. T. Abburi V. L. Alvarado A. Saminathan T. Vajja V. G. (2016). Genome-wide differentiation of various melon horticultural groups for use in GWAS for fruit firmness and construction of a high resolution genetic map. *Front. Plant Sci.* 7:e1437. 10.3389/fpls.2016.01437 27713759 PMC5031849

[B33] Parralondono S. Fiedler K. Kavka M. Samans B. Wieckhorst S. Zacharias A. (2018). Genetic dissection of early-season cold tolerance in sorghum: genome-wide association studies for seedling emergence and survival under field and controlled environment conditions. *Theor. Appl. Genet.* 131 581–595. 10.1007/s00122-017-3021-2 29147737

[B34] Paull R. (1990). “Chilling injury of crops of tropical and subtropical origin,” in *Chilling Injury of Horticultural Crops* eds Wang C. Y. (Boca Raton, FL: CRC Press), 7–36.

[B35] Pitrat M. (2008). “Melon,” in *Handbook of Plant Breeding* Vol. 1 eds Prohens J. Nuez F. (New York, NY: Springer), 283–315.

[B36] Pitrat M. (2013). Phenotypic diversity in wild and cultivated melons (*Cucumis melo*). *Plant Biotechnol.* 30 273–278. 10.5511/plantbiotechnology.13.0813a

[B37] Pritchard J. K. Stephens M. Donnelly P. (2000). Inference of population structure using multilocus genotype data. *Genetics* 155 945–959. 10835412 10.1093/genetics/155.2.945 PMC1461096

[B38] Revilla P. Rodríguez V. M. Ordás A. Rincent R. Charcosset A. Giauffret C. (2016). Association mapping for cold tolerance in two large maize inbred panels. *BMC Plant Biol.* 16:e127. 10.1186/s12870-016-0816-2 27267760 PMC4895824

[B39] Schläppi M. R. Jackson A. K. Eizenga G. C. Wang A. Chu C. Shi Y. (2017). Assessment of five chilling tolerance traits and GWAS mapping in rice using the USDA mini-core collection. *Front. Plant Sci.* 8:e957. 10.3389/fpls.2017.00957 28642772 PMC5463297

[B40] Semeniuk P. Moline H. E. Abbott J. A. (1986). A comparison of the effects of ABA and an antitranspirant on chilling injury of coleus, *Cucumbers*, and *Dieffenbachia*. *J. Am. Soc. Hortic. Sci.* 111 241–257.

[B41] Shi Y. Ding Y. Yang S. (2015). Cold signal transduction and its interplay with phytohormones during cold acclimation. *Plant Cell Physiol.* 56 7–15. 10.1093/pcp/pcu115 25189343

[B42] Sun Y. Xu Y. Peng J. Zhou M. Li Y. Li A. (2004). Screening on identification indices of chilling tolerance in melon. *China Veget.* 4 7–10.

[B43] Tamura K. Stecher G. Peterson D. Filipski A. Kumar S. (2013). MEGA6: molecular evolutionary genetics analysis version 6.0. *Mol. Biol. Evol.* 30 2725–2729. 10.1093/molbev/mst197 24132122 PMC3840312

[B44] Thomashow M. F. (2010). Molecular basis of plant cold acclimation: insights gained from studying the CBF cold response pathway. *Plant Physiol.* 154 571–577. 10.1104/pp.110.161794 20921187 PMC2948992

[B45] Tomason Y. Nimmakayala P. Levi A. Reddy U. (2013). Map-based molecular diversity, linkage disequilibrium and association mapping of fruit traits in melon. *Mol. Breed.* 31 829–841. 10.1007/s11032-013-9837-9

[B46] Wang Y. Xu Y. Li Q. Wang P. Hu J. Yang L. (2017). Discovery of related locus on core collection of melon (*Cucumis melo*) fruit character based on GWAS. *J. Agric. Biotech.* 25 1434–1442. 10.3969/j.issn.1674-7968.2017.09.006

[B47] Wang Z. D. Wu J. X. (2014). *Research on the Development of Muskmelon Industry Economy in China.* Beijing: China Social Science Press.

[B48] Xu Y. Wang Y. Hu J. Yang L. Li Q. Sun S. (2017). Association mapping of soluble solid content in melon fruits and exploration of the elite alleles. *Acta Hortic. Sin.* 44 90–910. 10.16420/j.issn.0513-353x.2016-0840

[B49] Yang L. (2007). *Studies on the Heat Tolerance and Gene of Heat Stress Response Related in Cucumber*. Nanjing: Nanjing Agricultural University Press.

[B50] Yan J. Warburton M. Crouch J. (2011). Association mapping for enhancing maize (*Zea mays L*.) genetic improvement. *Crop Sci.* 51 433–449. 10.2135/cropsci2010.04.0233

[B51] Zhang B. Shi W. Li W. Chang X. Jing R. (2013). Efficacy of pyramiding elite alleles for dynamic development of plant height in common wheat. *Mol. Breed.* 32 327–338. 10.1007/s11032-013-9873-5 23976874 PMC3748324

[B52] Zhang M. Ye J. Xu Q. Feng Y. Yuan X. Yu H. (2018). Genome-wide association study of cold tolerance of Chinese indica rice varieties at the bud burst stage. *Plant Cell Rep.* 37 529–539. 10.1007/s0029 29322237

[B53] Zhang Q. Chen Q. Wang S. Hong Y. Wang Z. (2014). Rice and cold stress: methods for its evaluation and summary of cold tolerance-related quantitative trait loci. *Rice* 7:e24. 10.1186/s12284-014-0024-3 25279026 PMC4182278

[B54] Zhang T. Che F. Zhang H. Pan Y. Xu M. Ban Q. (2017). Effect of nitric oxide treatment on chilling injury, antioxidant enzymes and expression of the CmCBF1 and CmCBF3 genes in cold-stored Hami melon (*Cucumis melo L.*) fruit. *Postharvest Biol. Tec.* 127 88–98. 10.1016/j.postharvbio.2017.01.005

[B55] Zhao C. Lang Z. Zhu J. (2015). Cold responsive gene transcription becomes more complex. *Trends Plant Sci.* 20 466–468. 10.1016/j.tplants.2015.06.001 26072094 PMC4536100

[B56] Zhao J. Artemyeva A. Del Carpio D. P. Basnet R. K. Zhang N. Gao J. (2010). Design of a *Brassica rapa* core collection for association mapping studies. *Genome* 53 884–898. 10.1139/G10-082 21076504

[B57] Zhou Y. Xu Y. Wang Y. Li Q. Hu J. (2017). Establishment of a comprehensive evaluation system for chilling tolerance in melon seedlings based on principal component analysis and cluster analysis. *Chin. Bull. Bot.* 52 520–529. 10.11983/CBB16138

[B58] Zhu H. Guo L. Song P. Luan F. Hu J. Sun X. (2016). Development of genome-wide SSR markers in melon with their cross-species transferability analysis and utilization in genetic diversity study. *Mol. Breed.* 36:153 10.1007/s11032-016-0579-3

