# Supplementary material for: Dissecting the Genetic Architecture of Melon Chilling Tolerance at the Seedling Stage by Association Mapping and Identification of the Elite Alleles
Source: Front Plant Sci. 2018 Oct 31;9:1577. doi: 10.3389/fpls.2018.01577 (PMC6220089; doi:10.3389/fpls.2018.01577)
Supplement: Supplementary file 2 [file Image_2.pdf]

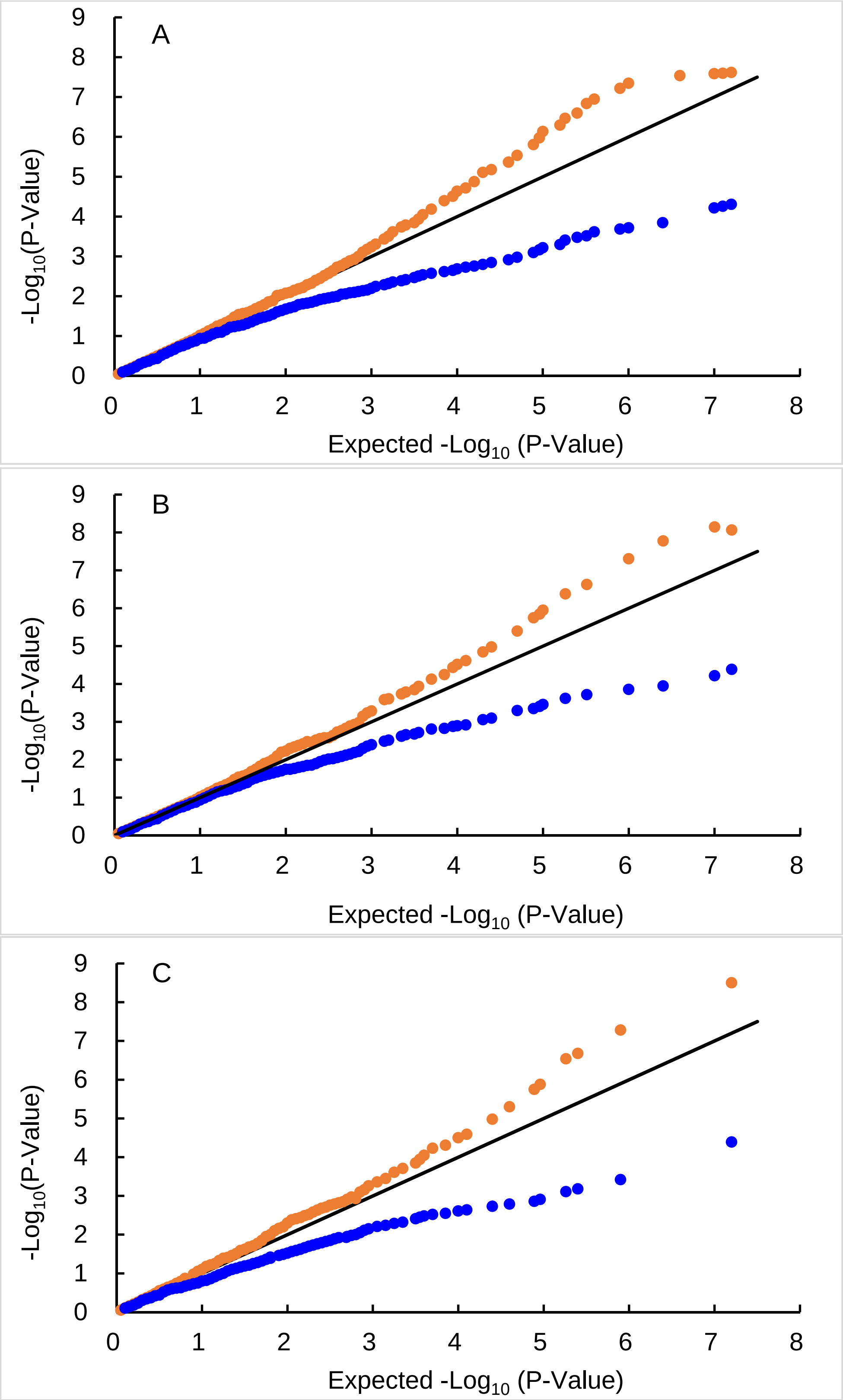

Figure S2 | Quantile-quantile plots of the GLM and MLM from association mapping in the whole panel (A), *melo* group (B), and *agrestis* group (C). ● GLM; ● MLM
