# Supplementary material for: Dissecting the Genetic Architecture of Melon Chilling Tolerance at the Seedling Stage by Association Mapping and Identification of the Elite Alleles
Source: Front Plant Sci. 2018 Oct 31;9:1577. doi: 10.3389/fpls.2018.01577 (PMC6220089; doi:10.3389/fpls.2018.01577)
Supplement: Supplementary file 3 [file Table_1.DOCX]

**TABLE S1** | The melon accessions used for association mapping and their chilling-injury indices (CIIs).

| No. | Accession | Subspecies^a^ | Origin | Seed source^b^ | CII | |
| --- | --- | --- | --- | --- | --- | --- |
|  |  |  |  |  | 2016 | 2017 |
| 1 | Baipikekouqi | *melo* | Sinkiang, China | NMGWM | 0.688 | 0.608 |
| 2 | Furong | *melo* | Sinkiang, China | NMGWM | 0.425 | 0.550 |
| 3 | Jinbangzi | *agrestis* | Sinkiang, China | NMGWM | 0.306 | 0.523 |
| 4 | Kalakekouqi | *melo* | Sinkiang, China | NMGWM | 0.519 | 0.558 |
| 5 | Bailangua | *melo* | USA | NMGWM | 0.854 | 0.645 |
| 6 | Manasiguadan | *melo* | Sinkiang, China | NMGWM | 0.500 | 0.540 |
| 7 | Baidonggua | *melo* | Sinkiang, China | NMGWM | 0.475 | 0.584 |
| 8 | Paodan | *melo* | Sinkiang, China | NMGWM | 0.415 | 0.504 |
| 9 | Baibadeng | *melo* | Sinkiang, China | NMGWM | 0.596 | 0.591 |
| 10 | 26332 | *melo* | Sinkiang, China | NMGWM | 0.458 | 0.413 |
| 11 | Lupisu | *melo* | Sinkiang, China | NMGWM | 0.577 | 0.588 |
| 12 | Zhongzhizhe | *melo* | Sinkiang, China | NMGWM | 0.291 | 0.333 |
| 13 | Lvpi-3 | *melo* | Sinkiang, China | NMGWM | 0.694 | 0.714 |
| 14 | Horneseoneen | *agrestis* | Henan, China | NMGWM | 0.310 | 0.400 |
| 15 | Honeymelon | *agrestis* | Guangdong, China | NMGWM | 0.400 | 0.380 |
| 16 | Baishami | *agrestis* | Jilin, China | NMGWM | 0.592 | 0.650 |
| 17 | Bingtangguan | *agrestis* | Henan, China | NMGWM | 0.575 | 0.570 |
| 18 | PI247894 | *melo* | The Soviet Union | NMGWM | 0.583 | 0.714 |
| 19 | PI438529 | *melo* | Taiwan, China | NMGWM | 0.444 | 0.556 |
| 20 | Xinshiji | *melo* | Japan | NMGWM | 0.729 | 0.626 |
| 21 | Xinyintian-16 | *melo* | Tunisia | NMGWM | 0.500 | 0.596 |
| 22 | Ezustanarst | *melo* | Hungary | NMGWM | 0.450 | 0.590 |
| 23 | Hamigua | *melo* | Japan | NMGWM | 0.458 | 0.588 |
| 24 | PI164610 | *melo* | India | NMGWM | 0.450 | 0.629 |
| 25 | Xianhuan | *agrestis* | South Korea | NMGWM | 0.449 | 0.550 |
| 26 | Kusong | *agrestis* | Malaysia | NMGWM | 0.450 | 0.559 |
| 27 | PI313970 | *melo* | The Soviet Union | NMGWM | 0.604 | 0.622 |
| 28 | PI1361951 | *melo* | Canada | NMGWM | 0.519 | 0.592 |
| 29 | PI163206 | *melo* | India | NMGWM | 0.583 | 0.600 |
| 30 | Danxingyinguo | *melo* | Bangladesh | NMGWM | 0.500 | 0.610 |
| 31 | PI164996 | *melo* | Turkey | NMGWM | 0.500 | 0.592 |
| 32 | PI177351 | *melo* | Turkey | NMGWM | 0.545 | 0.586 |
| 33 | Cdeenfcexheal | *melo* | Japan | NMGWM | 0.675 | 0.625 |
| 34 | PI171599 | *melo* | Turkey | NMGWM | 0.667 | 0.579 |
| 35 | Keorumekonob | *melo* | Japan | NMGWM | 0.479 | 0.587 |
| 36 | PI136214 | *melo* | Canada | NMGWM | 0.725 | 0.614 |
| 37 | PI381765 | *melo* | India | NMGWM | 0.345 | 0.418 |
| 38 | Huangdanzi | *melo* | the former Soviet Union | NMGWM | 0.625 | 0.569 |
| 39 | Aizi | *melo* | Sinkiang, China | NMGWM | 0.500 | 0.590 |
| 40 | Bayixiangli | *melo* | Sinkiang, China | NMGWM | 0.479 | 0.502 |
| 41 | Qiaerkehong | *melo* | Sinkiang, China | NMGWM | 0.525 | 0.571 |
| 42 | Kangbaier | *melo* | Sinkiang, China | NMGWM | 0.455 | 0.554 |
| 43 | Wangwentiangua | *melo* | Henan, China | NMGWM | 0.528 | 0.600 |
| 44 | Xunjin-1 | *melo* | Shaanxi, China | NMGWM | 0.472 | 0.750 |
| 45 | Hetaodonggua | *melo* | Inner Mongolia, China | NMGWM | 0.667 | 0.583 |
| 46 | Yinhui | *melo* | Taiwan, China | NMGWM | 0.340 | 0.400 |
| 47 | Yetiangua-1 | *agrestis* | Sinkiang, China | NMGWM | 0.450 | 0.525 |
| 48 | Xiaocuigua | *agrestis* | Hebei, China | NMGWM | 0.525 | 0.520 |
| 49 | Hongchengcui | *agrestis* | Inner Mongolia, China | NMGWM | 0.483 | 0.610 |
| 50 | Primo-4 | *melo* | USA | NMGWM | 0.500 | 0.564 |
| 51 | Huangxianggua | *agrestis* | Hunan, China | NMGWM | 0.500 | 0.650 |
| 52 | PI164409 | *melo* | India | NMGWM | 0.479 | 0.575 |
| 53 | PI169320 | *melo* | Turkey | NMGWM | 0.528 | 0.592 |
| 54 | PI164569 | *melo* | the former Soviet Union | NMGWM | 0.409 | 0.603 |
| 55 | Bajiang-2 | *agrestis* | Liaoning, China | NMGWM | 0.283 | 0.340 |
| 56 | Flower Fairy | *agrestis* | Beijing, China | NMGWM | 0.450 | 0.500 |
| 57 | TM-1 | *agrestis* | Guangxi, China | NMGWM | 0.575 | 0.650 |
| 58 | Pinghubaili | *agrestis* | Zhejiang, China | NMGWM | 0.492 | 0.650 |
| 59 | Yixuan-2 | *melo* | Guangdong, China | NMGWM | 0.560 | 0.600 |
| 60 | Yinxuanhuangzi | *agrestis* | Henan, China | NMGWM | 0.575 | 0.588 |
| 61 | Kangua | *agrestis* | Jiangsu, China | NMGWM | 0.619 | 0.600 |
| 62 | Wuyinxuan-1 | *agrestis* | Henan, China | NMGWM | 0.442 | 0.610 |
| 63 | Jiangxi Ligua | *agrestis* | Jiangxi, China | NMGWM | 0.550 | 0.650 |
| 64 | Tiancaigua | *agrestis* | Zhejiang, China | NMGWM | 0.400 | 0.433 |
| 65 | Huangjingua | *agrestis* | Shanghai, China | NMGWM | 0.605 | 0.608 |
| 66 | Baitangguan | *agrestis* | Heilongjiang, China | NMGWM | 0.650 | 0.608 |
| 67 | Yiwohou | *agrestis* | Shanxi, China | NMGWM | 0.567 | 0.650 |
| 68 | Yangjiaomi | *agrestis* | Shandong, China | NMGWM | 0.650 | 0.522 |
| 69 | Xiaohuadao | *agrestis* | Hebei, China | NMGWM | 0.588 | 0.608 |
| 70 | Shitiaoxian | *agrestis* | Shaanxi, China | NMGWM | 0.500 | 0.508 |
| 71 | Huangcaigua | *agrestis* | Jilin, China | NMGWM | 0.293 | 0.350 |
| 72 | Baiyingua | *agrestis* | Shandong, China | NMGWM | 0.475 | 0.500 |
| 73 | Xiguahun | *agrestis* | Henan, China | NMGWM | 0.567 | 0.550 |
| 74 | Huishuzi | *agrestis* | Liaoning, China | NMGWM | 0.608 | 0.550 |
| 75 | Qingpicui | *agrestis* | Liaoning, China | NMGWM | 0.400 | 0.408 |
| 76 | Denglonghong | *agrestis* | Henan, China | NMGWM | 0.600 | 0.750 |
| 77 | Jindaozi | *agrestis* | Liaoning, China | NMGWM | 0.500 | 0.643 |
| 78 | Sweet Banana | *agrestis* | Liaoning, China | NMGWM | 0.542 | 0.610 |
| 79 | Xiguahong | *agrestis* | Jilin, China | NMGWM | 0.541 | 0.608 |
| 80 | Qingdanzi | *agrestis* | Henan, China | NMGWM | 0.422 | 0.463 |
| 81 | Xiangmiangua | *agrestis* | Anhui, China | NMGWM | 0.543 | 0.608 |
| 82 | Red Seed Matisu | *agrestis* | Jiangsu, China | NMGWM | 0.546 | 0.600 |
| 83 | Portlro Gold | *agrestis* | South Korea | NMGWM | 0.406 | 0.442 |
| 84 | Jingnong-4 | *agrestis* | Hubei, China | NMGWM | 0.444 | 0.567 |
| 85 | Hebei melon | *agrestis* | Hebei, China | NMGWM | 0.426 | 0.525 |
| 86 | Ten Fluted Jingua | *agrestis* | Zhejiang, China | NMGWM | 0.422 | 0.505 |
| 87 | Jinbachi | *agrestis* | Inner Mongolia, China | NMGWM | 0.550 | 0.550 |
| 88 | Putian-1 | *agrestis* | Fujian, China | NMGWM | 0.525 | 0.525 |
| 89 | Hongzicui | *agrestis* | Henan, China | NMGWM | 0.525 | 0.567 |
| 90 | Hongpicui | *agrestis* | Jiangsu, China | NMGWM | 0.525 | 0.579 |
| 91 | Huahamapi | *agrestis* | Anhui, China | NMGWM | 0.492 | 0.550 |
| 92 | Niujiaomi | *agrestis* | Henan, China | NMGWM | 0.533 | 0.608 |
| 93 | Xiaolenggua | *agrestis* | Jiangsu, China | NMGWM | 0.450 | 0.525 |
| 94 | Yangshuigua | *agrestis* | Jiangsu, China | NMGWM | 0.433 | 0.579 |
| 95 | Tianedan | *agrestis* | Shanxi, China | NMGWM | 0.335 | 0.525 |
| 96 | Baiyugua | *agrestis* | Henan, China | NMGWM | 0.550 | 0.483 |
| 97 | Canta loupe chaudo | *melo* | USA | NMGWM | 0.400 | 0.371 |
| 98 | Hxle'sBese | *melo* | USA | NMGWM | 0.458 | 0.500 |
| 99 | Shushugua | *agrestis* | Shanxi, China | NMGWM | 0.345 | 0.410 |
| 100 | Xiehuatian | *agrestis* | Henan, China | NMGWM | 0.500 | 0.450 |
| 101 | Zhalaapan | *melo* | the former Soviet Union | NMGWM | 0.656 | 0.750 |
| 102 | Tur.82.1 | *melo* | Japan | NMGWM | 0.572 | 0.643 |
| 103 | PI251778 | *melo* | the former Soviet Union | NMGWM | 0.604 | 0.708 |
| 104 | NaelTukauie | *melo* | France | NMGWM | 0.469 | 0.500 |
| 105 | PI234607 | *melo* | South Africa | NMGWM | 0.389 | 0.375 |
| 106 | Xinyintian-13 | *melo* | Japan | NMGWM | 0.375 | 0.563 |
| 107 | Meiguobailangua | *melo* | USA | NMGWM | 0.361 | 0.575 |
| 108 | Kurumeke No6 | *melo* | Japan | NMGWM | 0.694 | 0.600 |
| 109 | Yinyintian-15 | *melo* | USA | NMGWM | 0.563 | 0.500 |
| 110 | Earls Fanaoito | *melo* | UK | NMGWM | 0.604 | 0.583 |
| 111 | West | *melo* | Sweden | NMGWM | 0.622 | 0.500 |
| 112 | B-18 | *melo* | Bangladesh | NMGWM | 0.650 | 0.750 |
| 113 | M510 | *melo* | Japan | NMGWM | 0.550 | 0.583 |
| 114 | Boduolige | *melo* | USA | NMGWM | 0.563 | 0.583 |
| 115 | PI165516 | *melo* | India | NMGWM | 0.563 | 0.625 |
| 116 | Green Banana | *agrestis* | Heilongjiang, China | NMGWM | 0.650 | 0.550 |
| 117 | Akewuluke | *melo* | the former Soviet Union | NMGWM | 0.625 | 0.500 |
| 118 | AuigauaSamais | *melo* | USA | NMGWM | 0.591 | 0.750 |
| 119 | PI145594 | *melo* | India | NMGWM | 0.500 | 0.563 |
| 120 | PMR-45 | *melo* | Japan | NMGWM | 0.450 | 0.583 |
| 121 | PI169329 | *melo* | Turkey | NMGWM | 0.500 | 0.450 |
| 122 | Cautor | *melo* | Heilongjiang, China | NMGWM | 0.675 | 0.750 |
| 123 | Fengli | *melo* | Beijing, China | NMGWM | 0.558 | 0.725 |
| 124 | Miangua | *agrestis* | Shandong, China | NMGWM | 0.208 | 0.458 |
| 125 | Jinfeng2610 | *melo* | Henan, China | NMGWM | 0.525 | 0.583 |
| 126 | Valley pac | *melo* | France | NMGWM | 0.220 | 0.400 |
| 127 | PI163208 | *melo* | India | NMGWM | 0.420 | 0.511 |
| 128 | Jinmandi | *agrestis* | Hong Kong, China | NMGWM | 0.275 | 0.442 |
| 129 | Elingbai | *agrestis* | Hebei, China | NMGWM | 0.542 | 0.650 |
| 130 | Yangsugua | *agrestis* | Henan, China | NMGWM | 0.316 | 0.495 |
| 131 | Jinmali | *agrestis* | Gansu, China | NMGWM | 0.273 | 0.317 |
| 132 | Baituwa | *agrestis* | Shaanxi, China | NMGWM | 0.567 | 0.483 |
| 133 | PI166190 | *melo* | India | NMGWM | 0.303 | 0.441 |
| 134 | Kalakesai | *melo* | Sinkiang, China | NMGWM | 0.475 | 0.567 |
| 135 | PI313973 | *melo* | Sinkiang, China | NMGWM | 0.492 | 0.531 |
| 136 | Houroudonggua | *melo* | Sinkiang, China | NMGWM | 0.500 | 0.567 |
| 137 | PI136192 | *melo* | Canada | NMGWM | 0.521 | 0.417 |
| 138 | Shanghaimelon | *agrestis* | Shanghai, China | NMGWM | 0.400 | 0.503 |
| 139 | Tyeha | *melo* | the former Soviet Union | NMGWM | 0.667 | 0.633 |
| 140 | PI293789 | *melo* | the former Soviet Union | NMGWM | 0.386 | 0.475 |
| 141 | Xinyiintian-19 | *melo* | Japan | NMGWM | 0.705 | 0.661 |
| 142 | Xinyiintian-17 | *melo* | Japan | NMGWM | 0.523 | 0.250 |
| 143 | Nafl-Tukauie | *melo* | France | NMGWM | 0.438 | 0.614 |
| 144 | 20KH.111 | *melo* | Afghanistan | NMGWM | 0.500 | 0.603 |
| 145 | Honeylew | *melo* | USA | NMGWM | 0.391 | 0.375 |
| 146 | PI276661 | *melo* | the former Soviet Union | NMGWM | 0.542 | 0.536 |
| 147 | EPRITEL | *melo* | Belgium | NMGWM | 0.646 | 0.594 |
| 148 | PI476338 | *melo* | the former Soviet Union | NMGWM | 0.464 | 0.500 |
| 149 | PI165452 | *melo* | Mexico | NMGWM | 0.722 | 0.643 |
| 150 | PI137852 | *melo* | Iran | NMGWM | 0.607 | 0.565 |
| 151 | PI1406371 | *melo* | Iran | NMGWM | 0.560 | 0.606 |
| 152 | PI140815 | *melo* | Iran | NMGWM | 0.667 | 0.591 |
| 153 | PI136206 | *melo* | Canada | NMGWM | 0.514 | 0.557 |
| 154 | PI163219 | *melo* | India | NMGWM | 0.617 | 0.750 |
| 155 | PI164852 | *melo* | India | NMGWM | 0.542 | 0.729 |
| 156 | Chaoxiangua | *agrestis* | Liaoning, China | NMGWM | 0.525 | 0.608 |
| 157 | White Banana | *agrestis* | Heilongjiang, China | NMGWM | 0.538 | 0.567 |
| 158 | Lanpang1902 | *melo* | Beijing, China | NMGWM | 0.727 | 0.568 |
| 159 | Meiganma241 | *melo* | Gansu, China | NMGWM | 0.688 | 0.615 |
| 160 | 92004 | *melo* | Iran | NMGWM | 0.625 | 0.593 |
| 161 | 93009 | *melo* | Taiwan, China | NMGWM | 0.396 | 0.429 |
| 162 | PI140678S1 | *melo* | Iran | NMGWM | 0.591 | 0.565 |
| 163 | PI260649 | *melo* | the former Soviet Union | NMGWM | 0.525 | 0.600 |
| 164 | PI136196 | *melo* | Canada | NMGWM | 0.525 | 0.636 |
| 165 | PI165508 | *melo* | India | NMGWM | 0.500 | 0.626 |
| 166 | PI164320S1 | *melo* | India | NMGWM | 0.667 | 0.601 |
| 167 | PI164569 | *melo* | India | NMGWM | 0.564 | 0.650 |
| 168 | Britianchueen | *melo* | UK | NMGWM | 0.618 | 0.688 |
| 169 | PI136213 | *melo* | Canada | NMGWM | 0.714 | 0.725 |
| 170 | PI143215 | *melo* | the former Soviet Union | NMGWM | 0.650 | 0.600 |
| 171 | PI169358 | *melo* | Turkey | NMGWM | 0.714 | 0.708 |
| 172 | EzuseananS2 | *melo* | Hungary | NMGWM | 0.571 | 0.750 |
| 173 | PI321004S2 | *melo* | Taiwan, China | NMGWM | 0.643 | 0.675 |
| 174 | PI143215(1) | *melo* | Iran | NMGWM | 0.750 | 0.500 |
| 175 | PI136223 | *melo* | Iran | NMGWM | 0.667 | 0.611 |
| 176 | Liujingua | *agrestis* | Shandong, China | NMGWM | 0.650 | 0.567 |
| 177 | Whitemelon | *agrestis* | Anhui, China | NMGWM | 0.525 | 0.483 |
| 178 | 337 | *melo* | Heilongjiang, China | NMGWM | 0.750 | 0.611 |
| 179 | 340 | *melo* | Heilongjiang, China | NMGWM | 0.536 | 0.618 |
| 180 | Ojin | *melo* | Beijing, China | NMGWM | 0.714 | 0.550 |
| 181 | Xuan'an87 | *agrestis* | Henan, China | NMGWM | 0.358 | 0.445 |
| 182 | Bailigua | *agrestis* | Hubei, China | NMGWM | 0.550 | 0.600 |
| 183 | Hamasu-5 | *agrestis* | Liaoning, China | NMGWM | 0.525 | 0.567 |
| 184 | Liu9602 | *melo* | Henan, China | NMGWM | 0.550 | 0.614 |
| 185 | Yinxiang | *melo* | Henan, China | NMGWM | 0.614 | 0.614 |
| 186 | Fengxuan-3 | *agrestis* | Henan, China | NMGWM | 0.622 | 0.543 |
| 187 | 875645 | *melo* | Henan, China | NMGWM | 0.500 | 0.361 |
| 188 | Xintiangua | *agrestis* | Henan, China | NMGWM | 0.558 | 0.511 |
| 189 | Fenghuang | *agrestis* | Taiwan, China | NMGWM | 0.525 | 0.437 |
| 190 | Elizabeth-239 | *agrestis* | Japan | NMGWM | 0.303 | 0.350 |
| 191 | Lutian-2 | *agrestis* | Shandong, China | NMGWM | 0.300 | 0.281 |
| 192 | Xiaohuanggua | *agrestis* | Henan, China | NMGWM | 0.543 | 0.483 |
| 193 | PI508450 | *melo* | South Korea | NGRP | 0.305 | 0.402 |
| 194 | Ames29858 | *melo* | France | NGRP | 0.348 | 0.441 |
| 195 | PI126156 | *melo* | Afghanistan | NGRP | 0.431 | 0.400 |
| 196 | PI508451 | *melo* | South Korea | NGRP | 0.407 | 0.349 |
| 197 | PI508447 | *melo* | South Korea | NGRP | 0.301 | 0.297 |
| 198 | PI282448 | *melo* | South Africa | NGRP | 0.444 | 0.419 |
| 199 | PI306131 | *melo* | Iran | NGRP | 0.577 | 0.643 |
| 200 | PI140755 | *melo* | Iran | NGRP | 0.355 | 0.380 |
| 201 | PI269353 | *melo* | Iran | NGRP | 0.528 | 0.607 |
| 202 | PI143231 | *melo* | Iran | NGRP | 0.514 | 0.600 |
| 203 | PI211853 | *melo* | Iran | NGRP | 0.407 | 0.402 |
| 204 | PI140662 | *melo* | Iran | NGRP | 0.522 | 0.525 |
| 205 | PI140815 | *melo* | Iran | NGRP | 0.349 | 0.408 |
| 206 | PI296385 | *melo* | Iran | NGRP | 0.422 | 0.420 |
| 207 | PI263397 | *melo* | Iran | NGRP | 0.647 | 0.558 |
| 208 | PI137834 | *melo* | Iran | NGRP | 0.523 | 0.541 |
| 209 | PI140766 | *melo* | Iran | NGRP | 0.487 | 0.455 |
| 210 | PI123689 | *melo* | India | NGRP | 0.571 | 0.620 |
| 211 | PI164328 | *melo* | India | NGRP | 0.400 | 0.416 |
| 212 | PI164400 | *melo* | India | NGRP | 0.525 | 0.546 |

^a^The classification of cultivar group is based on the criterion reported by Pitrat (2008).

^b^*NGRP* National Genetic Resources Program, USDA-ARS; *NMGWM* National Mid-termGenebank for Watermelon and Melon (Zhengzhou, China).
