# Supplementary material for: Dissecting the Genetic Architecture of Melon Chilling Tolerance at the Seedling Stage by Association Mapping and Identification of the Elite Alleles
Source: Front Plant Sci. 2018 Oct 31;9:1577. doi: 10.3389/fpls.2018.01577 (PMC6220089; doi:10.3389/fpls.2018.01577)
Supplement: Supplementary file 4 [file Table_2.DOCX]

**TABLE S2** | The information of the 272 SSR markers used in the present study.

| No. | Marker name | Chr. | Position/Mb | Forward primer | Reverse Primer | Source |
| --- | --- | --- | --- | --- | --- | --- |
| 1 | SSR008408 | 1 | 0.150 | CAACTCTCCACCATTGCTCA | TTCTTTTTAGATCCAACGCCT | ZH |
| 2 | CM07 | 1 | 0.860 | TTTCCCGCATTGATTTTCTC | GAGAAACGCTTCCCACAAAC | DL |
| 3 | SSR008659 | 1 | 1.730 | CCTCTTCCTTGCACCAACAT | ATGGAAAAGCGGGTAGAGGT | ZH |
| 4 | HNM10 | 1 | 2.810 | TCCAACCTCATCTTCCCCTC | TTGATTTAGATGAAAACCCAGC | DL |
| 5 | CMCTN86 | 1 | 4.400 | AAGGGAATGCATGTGGAC | GTGACAGTTATCAAGGATGC | DL |
| 6 | CM26 | 1 | 5.360 | CCCTCGAGAAACCAGCAGTA | CACCTCCGTTTTTCATCACC | DL |
| 7 | SSR009175 | 1 | 6.030 | CGACAATAGTCGTTTGCTATCAC | GGATCGTTACACTTTTTAGCCC | ZH |
| 8 | SSR009343 | 1 | 8.235 | TCATTGAACGCATGTTGGTC | CGGATTATCACATGAGAACCATT | ZH |
| 9 | SSR009556 | 1 | 9.813 | AATACAGGAAACGCATTGGC | TCGCCAATAGAAAGGGATTG | ZH |
| 10 | SSR009461 | 1 | 10.993 | CTCGCTGGAATCCAATTCAT | CAAGACCACAACAAAAACCCA | ZH |
| 11 | SSR009617 | 1 | 12.060 | CCACGTTCCACATCAACAAC | GGTACGGGTCTTTTTCTTGGA | ZH |
| 12 | SSR009817 | 1 | 14.620 | TTTTACGTTGTGGGCTTGTG | AACAGCTAGAAGGGTGGGGT | ZH |
| 13 | CMCT505 | 1 | 16.530 | GACAGTAATCACCTCATCAAC | GGGAATGTAAATTGGATATG | DL |
| 14 | SSR010144 | 1 | 18.140 | TTAAGATGCCTTTGATGGAGC | TTTTGTGGAAACTGGAAGGG | ZH |
| 15 | CMCCA145 | 1 | 19.895 | GAGGGAAGGCAGAAACCAAAG | GCTACTTTTGTGGTGGTGG | DL |
| 16 | SSR010488 | 1 | 22.180 | TTGGCTCAATTGCAAGACAC | ACTCCTGACGCATGACCTTC | ZH |
| 17 | SSR010675 | 1 | 24.420 | ACCACCACATGATGATCCCT | AAAGCGCAAAGAAAGCCATA | ZH |
| 18 | SSR010787 | 1 | 25.770 | TCTCATGCGTCTCATTGCTC | TGGACAAAAGTTGAGGGAGAA | ZH |
| 19 | SSR010993 | 1 | 28.030 | GCCTTTCCTCAGTCATCAGG | TTTTTGTTTGGAAGGGACAGA | ZH |
| 20 | SSR011330 | 1 | 30.630 | ACCCATATCCAACTCTCCCC | TGAAGAAATGGGTTTGGAGG | ZH |
| 21 | SSR011384 | 1 | 31.240 | GCACCCAAACGTTGCTCTAT | CCACTCCACCCACTACACCT | ZH |
| 22 | CMTTAAN244 | 1 | 32.320 | CTAAAGCTCTAAATGAAATCG | CAAAGCCTAAAGAGATTCG | DL |
| 23 | HNM7 | 1 | 33.190 | TATCGCAACGTCTGTTACTTCTC | GATTTCCTTCCGATTCCTTCA | DL |
| 24 | CMCTN4 | 1 | 34.684 | AAAACAAAAGCTCTCCACGA | CTTTCCTTTATTATGCCTACG | DL |
| 25 | SSR011967 | 1 | 34.950 | GTCTTCGCCATGCTTCTCTC | AATCGGAGAAGCACAACAGC | ZH |
| 26 | HNM15 | 2 | 0.180 | CGACCTTCATCACTACCCATTACT | TGTTGTTCGTTCGTCTGCCA | DL |
| 27 | CMGA36 | 2 | 0.901 | TACATTATGGGTAAGGTAAG | CCATCTCTTAACTTTCTCTC | DL |
| 28 | CmSUS1 | 2 | 2.785 | CCTGCAACATCATGAACTG | CTCTCTCTAGGCCAATCTCC | DL |
| 29 | ECM61 | 2 | 3.310 | TTTCAAAAAGCGAACCAGCTA | TCGGACTCGATTACCAAACA | DL |
| 30 | SSR012562 | 2 | 4.140 | TGTTTTCATAGGGGACCTGG | AACATGGCTAAGAGCAGCGT | ZH |
| 31 | gSSR4946 | 2 | 6.211 | AACCCTAACTCTAGTGAACCAA | CAACGATGTTAGTTTTTCCAC | CM |
| 32 | gSSR4947 | 2 | 6.226 | ATGAGGGCCCAGTATAGATAA | AAACACCAAAAGAGGAGTTATG | CM |
| 33 | gSSR4948 | 2 | 6.240 | TATGAGATTGAAGTCCGATCA | TTCGAAACTAACCTTTTGTTG | CM |
| 34 | gSSR4949 | 2 | 6.241 | CCTTCTGTGAATAAGTTCGTG | AAGTCTATCAATGTCTATCATCG | CM |
| 35 | gSSR4950 | 2 | 6.248 | TCTACCTCACAGTTTGTCGAT | GATGCCGTAAATTTGATTCTA | CM |
| 36 | gSSR4951 | 2 | 6.306 | ACCTTTACGTCGAGAGAAAAT | CTTTATTTGATGTGTGCGAAT | CM |
| 37 | gSSR4952 | 2 | 6.317 | TCTTTCACTCTCTCCAATCTCT | CACAGTTTCTGAACTTTCCAT | CM |
| 38 | gSSR4954 | 2 | 6.328 | ATGATTTGATTGCAGGATATG | GCAATAAGTGTTTTGATTTAGTG | CM |
| 39 | gSSR4955 | 2 | 6.334 | CCAAACATGAAGAATCTGAAC | TCAAGGTTCTTCTCCTTTTCT | CM |
| 40 | gSSR4958 | 2 | 6.424 | TGACAATTTGAAAGAGATTGG | GAGGTTAGGCAATCAAGCTAT | CM |
| 41 | gSSR4959 | 2 | 6.428 | AACCCAAACTCAAACTCAAAC | GGGAGGAAGAAGAGGAAATA | CM |
| 42 | gSSR4960 | 2 | 6.457 | CAAAAATTGGGATGTTGATT | GGGTTGACCTACTATCCTCTG | CM |
| 43 | SSR012892 | 2 | 8.550 | TCCCGCCTGCTTAAAACTAA | GCGGGTCATGACAAAAGGTA | ZH |
| 44 | SSR013073 | 2 | 11.740 | TCCATAAACATTTCCCCGTT | GTGCAAATGCAAGATCGAAG | ZH |
| 45 | SSR013163 | 2 | 13.250 | AAATTGGAGGAGGAGGAGGA | CAACAAAACCGCCTCAAAAT | ZH |
| 46 | TJ24 | 2 | 15.385 | AAACACGGGCTTGAAGAAAA | CCCAGAAGGTGAGAGAGACCT | DL |
| 47 | SSR013487 | 2 | 16.440 | CTGCCCCTTTTTCCTTCTCT | ATTGGCCTTTGTTTTCATGG | ZH |
| 48 | SSR013660 | 2 | 18.240 | TGGGATGGGACCAATAGTGT | AAGGGCGTTAGTTGAGGGAT | ZH |
| 49 | CMGA108 | 2 | 20.080 | CTCCTTCAAACATTGTGTGTG | GAGATAGGTATAGTATAGGGG | DL |
| 50 | CMBR066 | 2 | 20.590 | TCAAGCAAAAACCATAATCAGAA | TCCCTTTTCATCATTTCTCTTCA | DL |
| 51 | SSR014120 | 2 | 22.000 | CCCTGCAAACTACCCATTCT | CGAATCGTCGTCAAAACAAA | ZH |
| 52 | DE1329 | 2 | 23.830 | AATGCCACCTTTTTACTCATC | AAACCAAACTGATTTCCCC | DL |
| 53 | SSR014540 | 2 | 24.440 | GACACATCTTTCCCACACCA | CACTGTTTTGGGTATGGCCT | ZH |
| 54 | SSR014594 | 3 | 0.690 | GAGAGCCTTGAAGCTGAGGA | CTCCCTTCACTCCAAATCCA | ZH |
| 55 | SSR014660 | 3 | 1.310 | CTTCTCACATGCTTCAACGC | CCCCATTCCCATTTCTTTTT | ZH |
| 56 | SSR014769 | 3 | 2.430 | TATGTCCTCTCTTGGTCGCC | AGTCGATTGGCAAATTACCG | ZH |
| 57 | CSWCT10 | 3 | 3.915 | AGATCGGAATTGAAAAAG | AAAGGGGCTTCCTCTCTA | DL |
| 58 | SSR014992 | 3 | 4.790 | CCCTATGGCATATCGTGGTC | GAGCACGCCAGTTTATTGGT | ZH |
| 59 | SSR015119 | 3 | 6.270 | TTCAACCCAAGCATTTAGCC | TCATGTGGAATGTTTGCTTTTC | ZH |
| 60 | SSR015284 | 3 | 8.440 | TCGAGCTTCTTTTCGATTTAGG | TCATCTCGGGATTCATACCA | ZH |
| 61 | SSR015399 | 3 | 10.020 | GAACATTTAACTATCGTGGTAGGC | TGTAAGGTTCCAAAGCCTCG | ZH |
| 62 | SSR015533 | 3 | 12.120 | TGCGACGAGTAGCAAGAGAA | TCCCTTTATCAGGGTTGCAC | ZH |
| 63 | SSR015603 | 3 | 13.550 | GCTCTCATTGCATCACCAAA | TTGGATTGAGAGGCAAAACC | ZH |
| 64 | SSR015784 | 3 | 16.680 | TTAATCTTGCGGTGGAAAGG | TTAGGGAAGGCAATCAATCG | ZH |
| 65 | SSR015829 | 3 | 17.050 | GGTTTGTGGCCTTGAAGATG | TCGCCAGTGTTAGCTTGTTG | ZH |
| 66 | SSR015928 | 3 | 18.080 | TCAACTCAACCCAACTCGAA | AGGGTTGTTGGGGTGTATTG | ZH |
| 67 | SSR016149 | 3 | 19.870 | TCAAGTTGACCCAAACCCTC | AACGCCCTGAGTAGTCTCCA | ZH |
| 68 | CMCTTN175 | 3 | 20.900 | CACGAGGCCCACACTGTG | GTGAGATTTTGACGATGAACC | DL |
| 69 | CMGA128 | 3 | 21.066 | ATGAAGAAGGGATATTCAAAG | ACTCCATTGTTGCTAACCTTT | DL |
| 70 | TJ31 | 3 | 21.475 | GAGGCCTCCTCAGCTCTACA | AGCCCATTAGCACAAGCTGA | DL |
| 71 | SSR016829 | 3 | 23.890 | CACCCTTCCTTACCAGGACA | GATGCTGTCTTTTTCGTCCC | ZH |
| 72 | CMCTN5 | 3 | 24.975 | CACCTTAAAGTTTAGCCCC | AAAAATGCAATGAACTGAGCGC | DL |
| 73 | DM0854 | 3 | 26.490 | GCACCCAAAATTGTAATGG | AGAAGGGATCAAAGTTAATATCAC | DL |
| 74 | TJ10 | 3 | 27.248 | ACGAGGAAAACGCAAAATCA | TGAACGTGGACGACATTTTT | ZH |
| 75 | SSR017422 | 4 | 0.270 | AGAAATGGGAAATGGGGTTC | TCAATGAAACAGTCCCCTCC | ZH |
| 76 | HNM33 | 4 | 1.050 | TTGCTTCTGCTTCGGACCTT | GGGAGATTTGAATGGTGGTG | DL |
| 77 | MU7667 | 4 | 2.430 | CGTCGAACACAAAATTTCCA | CGCATATTCCTCCAAATCCA | DL |
| 78 | CMTAN142 | 4 | 3.950 | CAGAGAAGCAAGTGCAGC | GATGAACAAGTCTTTCTAGCC | DL |
| 79 | SSR018045 | 4 | 4.030 | CCCCTTCTCTTCCATTCCTC | GCACAAAATTAAAGTGCCTTCG | ZH |
| 80 | SSR018207 | 4 | 6.200 | TGCCATGTGATGAGAAGCTA | CCTTTCCCAACATTTTCCCT | ZH |
| 81 | SSR018393 | 4 | 8.800 | CAATGCGATCATTTAACAATGC | TGGTTTTTACCAAAGTGGCA | ZH |
| 82 | SSR018564 | 4 | 11.430 | CCATATGAAAATTCAACTCTGACC | TGGTAGAGCGAAAGAAAGTTGA | ZH |
| 83 | HNM12 | 4 | 13.370 | ATCTTCTGTCTTGGCCTCCG | GAAAGGGGATGAGTAAAAGTTGAG | DL |
| 84 | SSR018835 | 4 | 14.430 | TCGATGGCCAACTCCTTTTA | TGGTGTCTAGCAGCCAAGG | ZH |
| 85 | SSR019034 | 4 | 16.390 | TGTGTGTGGATGGCACTTTT | GGAGAGACAACCCCAACAAA | ZH |
| 86 | SSR019195 | 4 | 18.570 | TGGTTAGAGAACATGTGTCATGC | CGATTTAAAACAAGCGTGAGAA | ZH |
| 87 | SSR019378 | 4 | 20.320 | TCGTTTAACAATGCGATCACT | GTGACGGAAAACGAAGAAGG | ZH |
| 88 | SSR019550 | 4 | 22.240 | CCGATAGATTAACCCTCAAGAA | TATTTTGGCGGTTGCAATTT | ZH |
| 89 | SSR019803 | 4 | 24.140 | CAAGCTCCCACCACCACTAT | GCAACTTATGGTTTGGGGAA | ZH |
| 90 | SSR020162 | 4 | 26.320 | GGGATCATGCATGGGTAAAC | AGCCCTCCAAATATGGCTAA | ZH |
| 91 | ECM134 | 4 | 27.700 | TCTTTCCTCTGCAAATCCTTCT | TGCTAAAGCTACATGCTGTCCT | DL |
| 92 | DE1810 | 4 | 29.480 | AAAAGAAGACAAGGAAAGCAG | TAATTAGGATGCCTTTGCC | DL |
| 93 | SSR020867 | 4 | 29.730 | CCACACGGACAAAAGCATAA | GTGATTGGAGTTTGACGGGT | ZH |
| 94 | SSR020947 | 5 | 0.100 | TTTACGCAAGGATGAAAGGG | AATGGGCGAATTAAGAAGCA | ZH |
| 95 | DE1035 | 5 | 1.070 | TCTTCATTTTCTTTGCCTTTC | CTTCCAAGGGTCTTCAATG | DL |
| 96 | CMAGN61 | 5 | 1.660 | GGAGACACAAGGAATATGTG | ATAACAAAGGGGCATAACAC | DL |
| 97 | DE1404 | 5 | 2.320 | AAAATCTCATTTACCATAAGATCC | GCAACACAATCTTTTGCAG | DL |
| 98 | SSR021507 | 5 | 4.300 | TGCCCCTCTCAAAACAATTC | CAAGTGTTGGGTGTGGTCAG | ZH |
| 99 | SSR021615 | 5 | 5.160 | TAGAAGAGGTATGGTGGCGG | AGAGATTCGAAAAGGGGGAA | ZH |
| 100 | ECM129 | 5 | 7.210 | TCAGACTCCATTTCAGAGCCTA | CTTCAACCCCATTTTCTCACA | DL |
| 101 | SSR021815 | 5 | 8.170 | TGGAACCCAAACAATCTGTG | AAGTCGTGGATTCACTCATCA | ZH |
| 102 | DE1557 | 5 | 10.830 | CAAAGACATAAGCCCGATG | AAAAGAAAGATACAAGTTAGGGC | DL |
| 103 | SSR022150 | 5 | 12.500 | GGTGGTGGAGAGTGTGGTTT | TCAGCCTCTACAATCACCCC | ZH |
| 104 | SSR022193 | 5 | 13.070 | CCAAAGGGCTACCAAGTGAA | TGTTGGGGGTTTGGAAATAA | ZH |
| 105 | SSR022432 | 5 | 16.940 | AGGCTGGCCGTTTATCAAAT | ACCCCAACCTCCAGATAAGG | ZH |
| 106 | SSR022483 | 5 | 17.730 | TTGTTGAAAGCCACCTAAGGA | CCGCTTTCTTTTTGAACTGC | ZH |
| 107 | CMGAN3 | 5 | 19.872 | GTTAAAGGCTATGGTATAGAAC | AGAATAAGGTCCACATAAGG | DL |
| 108 | SSR022672 | 5 | 20.320 | CCCTTTTGGCCTCAAAGTTA | TTGGGAGAATCACAAAAGAGG | ZH |
| 109 | CMATTN29 | 5 | 23.528 | GAGCAGCAAACACTGGAGATA | GCCTTCCCAAGATGCCATTAA | DL |
| 110 | SSR023138 | 5 | 24.980 | GGAGGACGAAAGACCAATGA | CGACCGCCATTAATCAAAAC | ZH |
| 111 | HNM16 | 5 | 26.980 | AGCGAAATGCTGAGGAGACA | AGAGGTAGCGTCGGTAGTGGT | DL |
| 112 | CMTAA166 | 5 | 28.181 | GAGAAAGAGATGGACAAATGG? | TCCGTCTACAAGCGTGACTGT | DL |
| 113 | SSR023709 | 6 | 0.170 | ATTAAGAGGCAACCCCTCGT | AGAAAGGGTAATGGATGGGG | ZH |
| 114 | SSR023969 | 6 | 1.720 | CCACATGTTGCCATCTATCG | TTGATTTCCAACGAACATCG | ZH |
| 115 | SSR024061 | 6 | 2.330 | TATGAAGGTCAGGTTTCCGC | AGTTCCTGCGAAGGATTGAA | ZH |
| 116 | SSR024227 | 6 | 3.330 | AATTGCCCCCAAAATCAAAT | AATCCCAACCATGTCAGAAC | ZH |
| 117 | HNM41 | 6 | 4.570 | TCTTTCCCATAATGGCCTCAG | AACTTGGCTTGGACAGGGAT | DL |
| 118 | DE1591 | 6 | 6.300 | AACTTTTCCCATTTCCGAC | TTTGTTACCTTAAATGATTGGTG | DL |
| 119 | SSR024947 | 6 | 7.440 | TCATCCACCTGTATGCCATT | TGCTTCTTCTGGTCAACCTTC | ZH |
| 120 | SSR025119 | 6 | 9.060 | GATTTGTTGGTTTGGTTGGG | TGGATTTGGTTGGATTTTGA | ZH |
| 121 | DE1103 | 6 | 12.970 | CACATGACTTTTCACAAACG | GAATTCTATCTCTGTCTATCAAAG | DL |
| 122 | CmUGP | 6 | 14.769 | ACGTTGGATGGCCGTTTGACAGGTTGTTAC | ACGTTGGATGTTTCGCCAGTTTTTTCCCAC | DL |
| 123 | SSR025633 | 6 | 16.240 | CAGAGCAGAATCGGAAGGAG | ACGGACCCAGAACACAACAT | ZH |
| 124 | SSR025828 | 6 | 18.790 | TTTCTTGTGGTGGGTTGTTG | ACCATCCATTCCAATTTCCA | ZH |
| 125 | SSR025990 | 6 | 21.240 | TTCCCTTTCATTTGTTGTTCC | TGCCAAGGTTCGAAAGAAAC | ZH |
| 126 | SSR026180 | 6 | 23.610 | TCGAAACCCCATAGCAGAAC | CCCCAAAGATTAAACCCCAT | ZH |
| 127 | SSR026426 | 6 | 25.840 | TGTGAACCCAAAATTTGCAG | TGGTGCCTTAAAATCAGCAAT | ZH |
| 128 | SSR026548 | 6 | 26.870 | ATCTCGCATGCTCAAGGAAT | TCATCAGTCAATGAAAACATCG | ZH |
| 129 | SSR026703 | 6 | 27.910 | TGGGAATGGATGTGATTTGA | GGAGTGAAAGGGAAATGCAA | ZH |
| 130 | CM30 | 6 | 29.390 | TCAAACCTAAACCCTAAACCTAACC | AGGATGATCGGGGAAGAAAT | DL |
| 131 | CMAGN52 | 6 | 31.706 | CCACCAACATAACACACAAC | CTCTCACACTGTTGGGAAGA | DL |
| 132 | CMCTN38 | 6 | 35.853 | TTCTGCATACCCTCTCCT | CACACTTCCAGATGGTTG | DL |
| 133 | SSR027037 | 7 | 0.420 | CCAATGCCTAACCTAACCGA | GAGAATGGAAGGAAAAGGGC | ZH |
| 134 | HNM45 | 7 | 0.830 | TTCCAACGAAATCCCACTGT | GACGGTTCTTGATGACGATG | DL |
| 135 | SSR027249 | 7 | 1.660 | TGGGAGTTCATTCTTTTGGG | GCGTTTTGTTGATGTGCTTG | ZH |
| 136 | CMAGN75 | 7 | 2.402 | TGGGTTTTCTTCTACTACTG | TGCTTTTACTCTCATTCAAC | DL |
| 137 | SSR027539 | 7 | 4.410 | TGTTGAAAAGAATGAAAGAGGG | GCAAGGCATCGACTAGGTTC | ZH |
| 138 | HNM26 | 7 | 5.360 | CCTTTTACCTTTTCCCCATTCC | CATCGGAGATAGTAATTCCAGCA | DL |
| 139 | SSR027834 | 7 | 7.820 | AAAGGACTGCCTTTTGGGTT | CGAAAGCTAAATAGAGATGAATTGG | ZH |
| 140 | SSR028015 | 7 | 9.810 | TCCGGAAAGTTGGGTCATTA | CGTTGTTGATGACCTGGTTG | ZH |
| 141 | gSSR22403 | 7 | 11.064 | TACGACTGAGATGAAGAGACG | GTTGAAAAAGGAAAAGGAAAG | CM |
| 142 | gSSR22404 | 7 | 11.067 | AAGTAACATAGTACACCCCATAA | TTTCTTTATCCCAATTGTGTG | CM |
| 143 | gSSR22405 | 7 | 11.072 | GGATGGAATACCCTCTGTAAC | ATTCTCAACCCAATCCATATT | CM |
| 144 | gSSR22406 | 7 | 11.077 | GAGGAAGATGAAGGTTGTTTT | AAGAAAAGGCAAAGGAAAAT | CM |
| 145 | gSSR22411 | 7 | 11.128 | TGGGTGATAGCTATTTTCCTT | TCAAAAACTTACCAAAAGTGG | CM |
| 146 | gSSR22413 | 7 | 11.162 | TATACGCAGCCACTTACTGAT | GTATTCTCAACATCCATGAGC | CM |
| 147 | gSSR22414 | 7 | 11.164 | ATAAAGTTGGTGGGACTTCAT | CTCATCTTGTAACCTCATTGC | CM |
| 148 | gSSR22415 | 7 | 11.165 | TACCGTAAATTTGTGGTGTTG | TGAAAATAAAACGGCTGACTA | CM |
| 149 | gSSR22416 | 7 | 11.197 | ACTAAATTGGATGAGGAAAT | CATTCTTAACTTTTCCCACAA | CM |
| 150 | gSSR22419 | 7 | 12.320 | TTTAGGGTTTAGGGGCTAATA | TTCATGAATTTAGGACACAT | CM |
| 151 | gSSR22457 | 7 | 13.078 | GGAGATTGTTCGGCTTAAATA | GGTCCTAATGGTCCTTTTTAT | CM |
| 152 | gSSR22459 | 7 | 13.098 | CTCACTTCAAAGCAAGTATGC | ATTGATAAGGAGGGAGATGAA | CM |
| 153 | gSSR22486 | 7 | 13.633 | TCATGTACAAGAATGGGTAGG | TCAACCCACTATAACCCACTA | CM |
| 154 | gSSR22487 | 7 | 13.794 | AAATGGATTAATGGTTTTGG | CTCCCGGTTGATACAATTAC | CM |
| 155 | gSSR22492 | 7 | 13.898 | AGAAATGACACAGCTTTCAAC | ACAAACTGGTTTTCTCGGTAT | CM |
| 156 | gSSR22493 | 7 | 13.901 | GCTATGAAAAGAGCATCTCAA | GCTTTCACTACTTATGCTGGA | CM |
| 157 | gSSR22494 | 7 | 13.906 | AACAATAACTCTTTCTACTTTCC | TCATGATCGAGTTTCTAGGC | CM |
| 158 | gSSR22495 | 7 | 13.949 | CATCCTCATCTTCTTCTCCTT | ACTGTGAGCTTCACACGAA | CM |
| 159 | gSSR22496 | 7 | 13.969 | TGATGGAAAATAGGAAAGAGA | AATGGAGGGGTAATCTTCTAA | CM |
| 160 | SSR028296 | 7 | 15.010 | TACTCAATTCCCCATCCCTC | TTTCCCAGCTTTTGTGGTTT | ZH |
| 161 | SSR028465 | 7 | 17.420 | GGCCAAAAACCATAATTACCC | TCAAGATGGGTCCCCAATTA | ZH |
| 162 | SSR028695 | 7 | 19.360 | AACCAAGAAGTTGGATAACCG | AGAATGGGTTGGAGGAACTTT | ZH |
| 163 | ECM182 | 7 | 21.006 | TTCTTCATAATTCTAAATTTTTCCATC | CCAGGTGGAAGTTTTGCTTC | DL |
| 164 | CMTCN30 | 7 | 21.903 | GGAGGGAAAGGAAAGAGAGA | GGCAAGAAGATGGCAAAGAT | DL |
| 165 | CMGAN21 | 7 | 22.520 | GCTGTAAAACGAAACGGAGA | CGATCTTCTTTATTCTTCGCC | DL |
| 166 | SSR029474 | 7 | 24.010 | AACCGCAAATACGAGACCTG | TCTCCTGCATAAACCCCAAG | ZH |
| 167 | CMGA15 | 7 | 25.410 | CGGCAAGACGATTGGCAGC | ATCACCGTAGCGAAGCACC | DL |
| 168 | CMGAN48 | 7 | 26.018 | TTTAGGTTACGAAAACCCAG | ACTTATTCTTCCCGAAAACG | DL |
| 169 | SSR029716 | 8 | 0.440 | GCGAAATGATTCCATGTTTG | TCCACTTGCTATCTTCTCTCCA | ZH |
| 170 | ECM88 | 8 | 1.149 | TGGGCCTACGCTACAAACTT | AGCAGCACAAAAGCACTTCA | DL |
| 171 | HNM2 | 8 | 1.800 | CTTTCAAGCCAAGCCATTCA | ACATTCTCGTCGTTGGGGAT | DL |
| 172 | SSR030193 | 8 | 2.890 | CCAGGGAGCTATTGATTCCA | ATGCGGATTCCAAGTTGTTC | ZH |
| 173 | SSR030317 | 8 | 3.510 | GCCTTGAGTGCTGGTTTCTC | TCATGGCTCAGCACTTCAAC | ZH |
| 174 | CMTAN151 | 8 | 4.330 | CGTGCATCGTGTAGAGAGC | GTGTGGCTTTAATTGTTTTTG | DL |
| 175 | HNM42 | 8 | 6.850 | GCCGTCGTTTCATTTCAGA | AAGTGGGTCAATTTACATTAGGT | DL |
| 176 | CMACC146 | 8 | 8.994 | CAACCACCGACTACTAAGTC | CGACCAAACCCATCCGATAA | DL |
| 177 | CMTCN56 | 8 | 10.513 | CTTTTCTCTTCTTCTATTCTC | ATCCAAAAGGAATCGGAAAG | DL |
| 178 | HNM31 | 8 | 11.150 | GCCGTTCTGTGCTTCTTCATT | TTCCGACTCCGTATGTTCAATC | DL |
| 179 | SSR031575 | 8 | 12.200 | AAATTCATCCATAAACGGCG | ATTTCAACCACCCGATTTCA | ZH |
| 180 | SSR031684 | 8 | 14.020 | AAAACTTGGTATGACCCCAGAA | AAAATATTCCCCGAATTGCC | ZH |
| 181 | SSR031821 | 8 | 16.340 | ACCACCCTAAACCGAACTCA | GGAAAATAGTTCAACCGTTGGT | ZH |
| 182 | SSR031956 | 8 | 18.380 | AAACACAGCGTCGAATACCC | CCCCATGAGGGAACTATGAA | ZH |
| 183 | HNM40 | 8 | 20.880 | TGCAATTAAAACTTCCACCAAA | TACAACCGTCGCCGTCCT | DL |
| 184 | CMAG59 | 8 | 22.526 | TTGGGTGGCAATGAGGAA | ATATGATCTTCCATTTCCA | DL |
| 185 | SSR032536 | 8 | 24.100 | AAAAGCAAATGGGTGAAACG | CAATAATTGGGATTTTGCGG | ZH |
| 186 | CMAT141 | 8 | 26.427 | AAGCACACCACCACCCGTAA | GTGAATGGTATGTTATCCTTG | DL |
| 187 | SSR032562 | 9 | 0.170 | ACCGGAAAGGAGCAGAAAAT | CCCCAAACACATACCAGGTC | ZH |
| 188 | SSR032698 | 9 | 1.000 | GCATGCCCTGAAAATTAAGC | ATTTGCCAAAACAAGGCAAG | ZH |
| 189 | CMTC47 | 9 | 1.699 | GCATAAAAGAATTTGCAGAC | AGAATTGAGAAGAGATAGAG | DL |
| 190 | SSR033066 | 9 | 3.990 | CTTTGCTCAAAAGGAGGCAC | CTCAAAGAATCCCACGAAGC | ZH |
| 191 | SSR033261 | 9 | 5.710 | GGTGCCGAACCTCACACTAT | AGGAAATAAAGGCCAACATCA | ZH |
| 192 | SSR033288 | 9 | 5.930 | TCGATCCACAGTGTCCAAAT | TTTCAGTTTCATTCTACGTTCGTC | ZH |
| 193 | SSR033431 | 9 | 7.990 | TGAAATTCATCGTCTGAGAGTACG | GAGGAGGAGGAGGAGGAAGA | ZH |
| 194 | SSR033639 | 9 | 11.460 | AGCAGTGGTAGCAGCAGTAGC | AAGTTTTTCCTTCCCCAGGA | ZH |
| 195 | SSR033748 | 9 | 12.620 | TGCACGTTTTGCAGCTCTAC | TCCACACGCTTTCACCATAA | ZH |
| 196 | SSR033838 | 9 | 14.000 | GAAATCACTTATCGACATAGTTCACTG | TCCTTGTTTTCGTGAGTTGCT | ZH |
| 197 | SSR033988 | 9 | 15.690 | TGCACGTTTTGCAGCTCTAC | TCCACACGCTTTCACCATAA | ZH |
| 198 | SSR034244 | 9 | 18.010 | TGTCATTCCAGTTATTCCATGC | TGGCACCTCGACGAATAAAT | ZH |
| 199 | SSR034430 | 9 | 19.620 | TTCGGTGACAGACGGTGATA | CTCTCCATGCATGCAACAAT | ZH |
| 200 | SSR034757 | 9 | 21.700 | TTGGCTCCTCTTAGTGTCCTC | ATGGTCTCCCCAACCTATCC | ZH |
| 201 | CMCTN7 | 9 | 22.071 | AATGACACTGCCCACATTCT | AGGTTTTTCAATGGAGGGGA | DL |
| 202 | CMATN22 | 9 | 23.381 | CGGCAATCATCTTATCTTTC | AAGATTGAAGTGGGAAAATG | DL |
| 203 | SSR035237 | 9 | 23.970 | GGTGGGAAGTTGTCTGCATT | GGTAGGAGTTTCGAGAGGGG | ZH |
| 204 | SSR035288 | 10 | 0.220 | ATTGGTGCCTATCCAATCCA | ATTCAACCCCTTTTCAACCC | ZH |
| 205 | SSR035466 | 10 | 1.330 | ACAATTCCAAAAGTGTGGGG | AGGAAATGGCATCAAACTCAA | ZH |
| 206 | CMCTN19 | 10 | 1.971 | GAATGATTGGAGCAACCAGT | GCTTTTTGAATTTGTGCAGGG | DL |
| 207 | CMTCN67 | 10 | 3.339 | TCTCTTACAACTCTTTGTCG | GGTTCAAGGATTCATCGTTG | DL |
| 208 | CMGA172 | 10 | 3.803 | CGGCAAGACGATTGGCAGC | ATCACCGTAGCGAAGCACC | DL |
| 209 | CM38 | 10 | 3.950 | TAGCATCTGATCGGAAAACC | CAACTTCATCCGCCAAGAAT | DL |
| 210 | HNM13 | 10 | 5.160 | GGCAGCCACTACTACCTCCTC | GCCACGACCATCAACCTTAC | DL |
| 211 | SSR036205 | 10 | 6.190 | GCAGCCCCAAAACATCTAAG | GTGCCTGCAAGCGTTTTATT | ZH |
| 212 | SSR036376 | 10 | 8.630 | GTGTCAAAATCGACCGACCT | GCTTTTTGGATGCTTTGTTGA | ZH |
| 213 | CMTCN8 | 10 | 9.993 | CCTCCGCCACATATTACAAT | TTCATCTTGACACGTAAGAG | DL |
| 214 | SSR036581 | 10 | 11.210 | TTCCCCCTTCATTCTCAAAT | GGAATAAACCTCATGTAGCCCA | ZH |
| 215 | SSR036720 | 10 | 13.390 | AAGATGGGTGTAAATGGTTGTT | GACACAAGACGTCGGGAAAT | ZH |
| 216 | DM0272 | 10 | 15.520 | TGATTATTTGCCCTTTAATTTAG | GTTTCAACGTCAACAGAGC | DL |
| 217 | SSR036925 | 10 | 16.180 | GCCAATTTACACCCTGCACT | TGCATCAATTCTTACCCGTG | ZH |
| 218 | HSSR007 | 10 | 17.318 | AAGGAAACTAGAACGTGGAAGC | CTCTGCGTATGAAGCAAACCAA | RG |
| 219 | HSSR008 | 10 | 17.574 | TGGGACAATGGAAGTTAAGTGG | TGGTGTTGTTCTGGTTTGACTT | RG |
| 220 | HSSR009 | 10 | 17.574 | GAGAAGTGACGATGACGAGGCT | TGTCTTCCTTAGAAAACGCCTT | RG |
| 221 | HSSR010 | 10 | 17.652 | GAGGTGGAGGATAAAACAAATG | GATGCTGATAGGAATACCGAGA | RG |
| 222 | HSSR006 | 10 | 20.579 | AGGAACAAAGTGCCCAAAATG | TAATCTTGAATGTTGCCGCTC | RG |
| 223 | HSSR005 | 10 | 20.579 | GAGGAGGAACAAAGTGCCCA | CAATCTTGAATGTTGCCGCTC | RG |
| 224 | HSSR004 | 10 | 20.579 | ACAGGAGGAGGAACAAAGTGCC | TCAATCTTGAATGTTGCCGCTC | RG |
| 225 | HSSR003 | 10 | 20.598 | TGCGATAAAGGATGTCGTGAAG | TGGATGGAAGAAAGAGGAAACC | RG |
| 226 | HSSR002 | 10 | 20.797 | TTGCCCCTAGTCAAAACTTATC | TGCCAGACTTTATGTGGACTTA | RG |
| 227 | HSSR001 | 10 | 20.804 | ATCGAAATCATCACTCAACCG | GTGTTGAAGTGCCAAAGTGTCT | RG |
| 228 | HSSR011 | 10 | 21.939 | AGATTAATAGGGCAACAAGAGC | ACTTCGTTCTATCTTCCCGTTA | RG |
| 229 | HSSR012 | 10 | 22.052 | GTCTGGCGAAGAAGGTGGTAAG | TTCCAACACTTCTCCAAATCGA | RG |
| 230 | HSSR013 | 10 | 22.090 | CGATGCGGAAGCTATCTATGAA | TTGAAAGCAGACGCAACATAAC | RG |
| 231 | HSSR014 | 10 | 23.745 | TGGTTTCTCGGTTCTGGTTGTA | CTCAGGGAATCTAGGAGAAGGG | RG |
| 232 | HSSR015 | 10 | 25.107 | GATGGTTGGCAAACGAGAAAT | TATGGAGTGAGGGGCCTAATG | RG |
| 233 | SSR036952 | 11 | 0.130 | CAATTTCCCCTTCTTCCCAT | TCGTCGTCATAGTCGTCGTC | ZH |
| 234 | DM0673 | 11 | 0.560 | TCTACGGACCATGTGAATC | TCAAACAAAGTTGAAATTAGG | DL |
| 235 | CMCT160a | 11 | 1.202 | GTCTCTCTCCCTTATCTTCCA | ACGGTGTTTGGTGTGAGAAG | DL |
| 236 | SSR037322 | 11 | 3.480 | ATAACAGAAGGCGCAAGGTG | TCAATTGGGCTCTCCTCATC | ZH |
| 237 | SSR037428 | 11 | 4.180 | TTTTTGGGTAGGGGAAATGT | TCACTCCGAGATCTTATAACCATT | ZH |
| 238 | SSR037596 | 11 | 6.010 | TCGTCCAATCAAATCAACCA | GGGAAAAGTTGGTTTTGGTG | ZH |
| 239 | DM0804 | 11 | 7.340 | GGAGTCCTTTTGCAACAG | CAAAAACTGATTGTTAACTTAAC | DL |
| 240 | SSR037766 | 11 | 8.510 | ATTTGCATTCACCCTTCACC | TGTATACAAATCTCCACACTCACATC | ZH |
| 241 | SSR037879 | 11 | 10.380 | AGGGAAATGTTTTGGTGCAG | CCTGGGCAAAGACAAAAGTC | ZH |
| 242 | SSR038098 | 11 | 13.880 | GAGATGTACCATTGTGGGGG | GCCTTATTCAAAACTGTTGGG | ZH |
| 243 | SSR038231 | 11 | 15.800 | TTCGAATCTTCATTTTAGCTCG | AATGGCACACATTGGGTTTT | ZH |
| 244 | SSR038372 | 11 | 17.080 | GAAAAGAGTTAGGCTTCCCAA | TCCATTCTCTCTTCCCTGGAT | ZH |
| 245 | SSR038578 | 11 | 19.580 | AAGGGAACAGACAATGAAGCA | AAGGCACTAAATTTCCTCATCTTG | ZH |
| 246 | SSR038670 | 11 | 20.340 | GAATAGTTTCGAATCTTTGGTGC | GGCTTTAGCCCTTTACCCCT | ZH |
| 247 | SSR038777 | 11 | 21.610 | TGGAAATTGGAAAACTTCGC | TGTCGTTTGGGTTCTCCTTC | ZH |
| 248 | SSR038830 | 11 | 22.070 | TGGTCATGGCCCCTAATCTA | TTCGATTTGCAAGAGACACG | ZH |
| 249 | CMBR071 | 11 | 23.900 | GCATACGACTCTTGGAAATCG | GAATCACGAAGAGAGCTGCAC | DL |
| 250 | MU44460 | 11 | 24.740 | TCCATTAATTCTTACGCGGC | TGAAGAGGAGGAATCGGAGA | DL |
| 251 | SSR039720 | 11 | 26.840 | AGAGTCTCATTCCTCTCGACG | TGTGTATGGAGGGCTTTTCC | ZH |
| 252 | DE1074 | 11 | 27.150 | AAGAAGTCCTGAGTGTGAGAG | CACCTCCTTCTTCATCTTCC | DL |
| 253 | CMGA104 | 11 | 28.771 | TTACTGGGTTTTGCCGATTT | AATTCCGTATTCAACTCTCC | DL |
| 254 | SSR039921 | 12 | 0.440 | GGAAGAAGGAGGATGGAAGG | TGTGGCGACCAACACACTAT | ZH |
| 255 | SSR040059 | 12 | 1.570 | TGGTCAAACAACATTTCCCC | GGGTGGGTACATTATTGAGATGA | ZH |
| 256 | DE1917 | 12 | 2.660 | TCCTAATACCTTTGAATTTTGC | AAGTTAATGCAACATCCTTTTG | DL |
| 257 | SSR040314 | 12 | 4.420 | TTTGCCACATCATCATGAAA | CCCATGTTTAAGTGATTCCCA | ZH |
| 258 | SSR040455 | 12 | 6.070 | GGTTGGGAGGTTTGTCAAGT | TCCATGAAACACAAGCAACC | ZH |
| 259 | SSR040634 | 12 | 8.730 | CACCAAGTGAAGGTGAAGCA | CACGTCAGCAACACCTGATT | ZH |
| 260 | SSR040765 | 12 | 10.490 | GTTGGTAGCATTTGTGGGCT | CCTTTTTCACTCTCCCTCCC | ZH |
| 261 | SSR040884 | 12 | 12.190 | GCTTAAGAGGAGGCAGGCTT | TCGGCCAAGAGACTCAGATT | ZH |
| 262 | CSWGAT01 | 12 | 13.508 | ACCCGTTCGTCTGTCTCT | CCGACCTCTAGGGGATAA | DL |
| 263 | SSR041025 | 12 | 14.590 | AAACCGAAAACCAAGATGGA | AATGGGGGTTTGTGTGAAAA | ZH |
| 264 | SSR041212 | 12 | 16.590 | AATGCAGGGACTGAATTTAAGA | TGTCCTCCAAGTTATGCCAA | ZH |
| 265 | SSR041311 | 12 | 17.750 | CTTCCAACATTCCATTGGCT | TGAAAAGAGATGGGAAACTTTAGTG | ZH |
| 266 | SSR041344 | 12 | 18.140 | CCATTCCTTTTGGTGCACTT | TGCATACTTCATTGGGGGTT | ZH |
| 267 | SSR041674 | 12 | 20.260 | TTCGAGGAAAAACAGATGGG | CAGCAAGCAGAAAAATGCAG | ZH |
| 268 | CMBR150 | 12 | 21.890 | TTTTTACTGTGTGTTTTGATTTGTT | TTGGTGGACTGGAATCCATA | DL |
| 269 | TJ29 | 12 | 22.690 | AGCCTAAGCCACCGATTTTT | TTCCCAAGTGGGGTTATGAG | DL |
| 270 | HNM38 | 12 | 23.840 | AATGGCGGCTTCAACTTAGA | GCATGGCTTCACAAATCTCAA | DL |
| 271 | SSR042363 | 12 | 24.700 | GGCTGTGCTTGGTTAGGTGT | TGTTGTGATGTGCATTAGGTGA | ZH |
| 272 | CMGAN80 | 12 | 25.797 | ATATTGATTGCTGGGAAAGG | CTTTTTTGGCTTTATTGGGTC | DL |

*CM*, *DL*, and *ZH* mean the markers collected from the database CmMDb (Chaduvula et al., 2015), the linkage map of Diaz et al. (2011), and the report of Zhu et al. (2016), respectively. *RG* means the markers developed by our research group.
