# Supplementary material for: Dissecting the Genetic Architecture of Melon Chilling Tolerance at the Seedling Stage by Association Mapping and Identification of the Elite Alleles
Source: Front Plant Sci. 2018 Oct 31;9:1577. doi: 10.3389/fpls.2018.01577 (PMC6220089; doi:10.3389/fpls.2018.01577)
Supplement: Supplementary file 5 [file Table_3.DOCX]

**TABLE S3** | The information of the 27 CAPS markers used in the present study.

| No. | Marker name | Chr. | Position/Mb | Forward primer | Reverse Primer | Enzyme |
| --- | --- | --- | --- | --- | --- | --- |
| 1 | M16H | 1 | 2.020 | TCCCAATAATCTGCAACCAAG | CCTACTTCAAACTGCTGCTCATA | HindⅢ |
| 2 | M14H | 1 | 17.078 | CAAATGTAGAGGACACAAGGAAGAT | AAGCAGGTGGTTCAGGAGGT | HindⅢ |
| 3 | M 28E | 2 | 12.939 | AGGCATACTATGAAGATGAGACCG | GGAATTTGGAGATGATGGGAGT | EcoRⅠ |
| 4 | M24P | 2 | 17.063 | TCACTCAACAACGAAACGCTTAC | GCTGCGACTGAAATGAAAGGTA | PstⅠ |
| 5 | M 3-1H | 3 | 2.935 | TTCCAAACCTCATCCCACTG | GACGAAACCCAATTACACCTCTA | HindⅢ |
| 6 | M34B | 3 | 5.578 | TGACCTCACTTCCATGTCTCTAGA | GCTTGTTCCTACGGCATCAC | BamHⅠ |
| 7 | M34P | 3 | 9.530 | TTGAACTGCTCCCAGATGATCT | GTGTTCCTATGGTCAAACCTATCC | PstⅠ |
| 8 | M4-1H | 4 | 0.342 | GTTTGGAAGATGACGTAGTTGACA | TTACGCAATAGTCGTTGGGATT | HindⅢ |
| 9 | M4-37P | 4 | 15.737 | TGTCTGTGCCACCATCTTATTC | CCGCCGTCTTCCTCATCTT | PstⅠ |
| 10 | M4-39H | 4 | 17.199 | CTCCTGTTGCCATGCTTGAT | TTCTCCTACCATGCTCATCTCA | HindⅢ |
| 11 | M4-3P | 4 | 19.006 | GGTCCCTCCCCAATTACTACTC | GCTATCGTGCTTGGAATCGTT | PstⅠ |
| 12 | M4-10E | 4 | 28.150 | TTTATCGAAATCGGTGGTGTG | AAGCTTTAGACTGGGCTTGGT | EcoRⅠ |
| 13 | M5-1B | 5 | 3.906 | AAGATCAACCTCCCTATATACGATG | CTGGACTTTTTGTTAGCTTCACTG | BamHⅠ |
| 14 | M5-12H | 5 | 25.238 | CCTTCCTCACTTCTCCACCTAA | CGATTCGTCGTGAAAGGGTAC | HindⅢ |
| 15 | M6-1H | 6 | 0.515 | GCCAGGGAGAGACGAGAATAG | CAGTAGGCGAATTAGCGGG | HindⅢ |
| 16 | M6-1B | 6 | 8.462 | CACAAACCAGAAGCCTGACAG | AGAAAGCCTTCATGGACACATT | BamHⅠ |
| 17 | M6-4P | 6 | 13.843 | TTGGATATGTAGGCACAACATCC | CTAGAATAGAATTGGGGCAACTGT | PstⅠ |
| 18 | M7-1H | 7 | 0.607 | CCATAAGACTGTTGATTTCGCA | TGATAACAACTCTACAGAGCCACC | HindⅢ |
| 19 | M8-1P | 8 | 7.822 | TACCCCTCAAGCAGACATTCA | CCCCATTGACATAATAGTTGCA | PstⅠ |
| 20 | M8-3E | 8 | 17.772 | ACTGAGCCCATACGCTACAGA | GTGGCAAGACTGTCGGAACTA | EcoRⅠ |
| 21 | M9-12P | 9 | 8.720 | TCCTGCCCATTTGCTCACT | TGATAGTCGCATCAAGGTCATTC | PstⅠ |
| 22 | M9-10E | 9 | 24.045 | GTTGATTCCACATCTCGGTAGT | ACACGATGCCATAAGATGAACA | EcoRⅠ |
| 23 | M10-1P | 10 | 4.498 | CCACGAACTGCTAAGGCTATTT | GTTGCCCCCATTATAAGGTG | PstⅠ |
| 24 | M11-9B | 11 | 9.186 | TCTGATACCAGCAACCTTGCT | TGCTGCTAGTCGTTACCCAAG | BamHⅠ |
| 25 | M11-13H | 11 | 16.972 | GCATTTAGGCAGTGGGTTTC | AATAGGGTTGACCTGGTGGG | HindⅢ |
| 26 | M11-14E | 11 | 18.299 | CACAAAAGACAACTCCCCATAA | GACTTCAGGTACATGCACAATACTG | EcoRⅠ |
| 27 | M12-11B | 12 | 15.929 | CAAGTCTTTCTTCGTGGCTTCT | TCAAATGGGGTTCAGCAAAT | BamHⅠ |
