# Supplementary material for: Dissecting the Genetic Architecture of Melon Chilling Tolerance at the Seedling Stage by Association Mapping and Identification of the Elite Alleles
Source: Front Plant Sci. 2018 Oct 31;9:1577. doi: 10.3389/fpls.2018.01577 (PMC6220089; doi:10.3389/fpls.2018.01577)
Supplement: Supplementary file 6 [file Table_4.DOCX]

**TABLE S4** | Candidate genes linked genomic region of the locus CMCT505 highly associated with chilling tolerance in melon.

| Code | Gene ID | Start (bp) | Stop (bp) | *Arabidopsis* homologues | Description | GO annotation |
| --- | --- | --- | --- | --- | --- | --- |
| 1 | MELO3C013397 | 16434115 | 16435317 | AT4G38840 | SAUR-like auxin-responsive family protein | response to auxin, response to cold |
| 2 | MELO3C013398 | 16438260 | 16438758 | AT4G38840 | SAUR-like auxin-responsive protein family | response to auxin, response to cold |
| 3 | MELO3C013399 | 16439963 | 16441126 | AT4G38840 | SAUR-like auxin-responsive protein family | response to auxin, response to cold |
| 4 | MELO3C013400 | 16443132 | 16443727 | AT4G38840 | SAUR-like auxin-responsive protein family | response to auxin, response to cold |
| 5 | MELO3C013401 | 16457883 | 16458790 | AT5G18030 | SAUR-like auxin-responsive protein family | response to auxin, auxin-activated signaling pathway, multicellular organism development, regulation of growth |
| 6 | MELO3C013402 | 16503909 | 16504694 | AT4G36110 | SAUR-like auxin-responsive family protein | response to auxin |
| 7 | MELO3C013403 | 16514817 | 16515618 | AT1G75590 | SAUR-like auxin-responsive protein family | response to auxin |
| 8 | MELO3C013404 | 16548027 | 16550298 | / | Cellulose synthase catalytic subunit [UDP-forming] | hydrogen ion transmembrane transporter activity |
| 9 | MELO3C013405 | 16553955 | 16554122 | / | Methionine-tRNA ligase | tRNA dihydrouridine synthase activity |
| 10 | MELO3C013406 | 16554648 | 16565596 | AT4G38890 | tRNA-dihydrouridine(47) synthase [NAD(P)(+)] | tRNA dihydrouridine synthase activity |
| 11 | MELO3C013407 | 16571554 | 16575224 | AT4G38900 | Putative transcription factor | transcription factor activity sequence-specific DNA binding |
| 12 | MELO3C013408 | 16584065 | 16588671 | AT4G38900 | Putative transcription factor PosF21 | transcription factor activity, sequence-specific DNA binding |
| 13 | MELO3C013409 | 16623603 | 16626822 | AT2G21240 | GAGA-binding transcriptional activator | GAGA-binding transcriptional activator |
| 14 | MELO3C013410 | 16633832 | 16637314 | AT2G21250 | NADP-dependent D-sorbitol-6-phosphate dehydrogenase | oxidoreductase activity |
| 15 | MELO3C013411 | 16642701 | 16649517 | AT2G21250 | NADP-dependent D-sorbitol-6-phosphate dehydrogenase | oxidoreductase activity |
